# Electrospinning of core shell nanofibers using amine modified sericins

**DOI:** 10.1038/s41598-025-08984-2

**Published:** 2025-12-01

**Authors:** Demet Sezgin Mansuroglu, Adem Çınarlı, Gökhan Çaylı, Demet Gurbuz

**Affiliations:** 1 Central Laboratory, Kocaeli University, 41001 Kocaeli, Türkiye; 2 Department of Chemistry and Chemical Processing Technologies, Kocaeli Vocational School, Kocaeli University, 41140 Kocaeli, Türkiye; 3 Department of Chemistry, Engineering Faculty, Istanbul University-Cerrahpaşa, 34320 Avcılar, Istanbul, Turkey; 4 Department of Engineering Sciences, Engineering Faculty, Istanbul University-Cerrahpaşa, Istanbul, Turkey

**Keywords:** Sericin modification, Electrospinning, Nucleophilic substitution, Nanofibers, Nanomaterials, Nanofibercomposites, Material characterization, Polymer chemistry, Materials science

## Abstract

In this study as a renewable resorce, silk sericin (SS), a protein surrounding fibroin fibers in silk, was chemically modified with various amines (e.g., methylamine, butylamine) to improve its functional properties. These modifications, which included nucleophilic substitution reactions, improved its thermal stability, mechanical strength, and biological activity by substituting primary amine groups in place of hydroxyl ions in the structure. Modified SS (M-SS) was then used to produce nanofiber membranes via coaxial electrospinning. In this process, polyvinyl alcohol (PVA) served as the hydrophilic and SS/M-SS carrier polymer to delay biodegradation, while hydrophobic polylactic acid (PLA) and polycaprolactone (PCL) formed the shell to enhance mechanical strength and provide minimum adhesion to wounded tissues. This technique enabled the stable fabrication of core–shell nanofibers containing SS/M-SS, which are typically difficult to electrospin alone. Among the M-SS variants, methylamine-modified sericin (SMAT) yielded nanofibers with the highest tensile strength (0.673 MPa) and 6.44% elongation. The highest thermal resistance was observed in methylamine (SNF7) and butylamine (SNF9) modifications, with SNF7 showing 5% mass loss at 250 °C. The lowest resistance was seen in benzylamine-modified fibers (SNF11) at 220 °C. SNF9 also exhibited the highest cell viability, indicating its potential for biomedical applications.

## Introduction

**Fig. 1:**
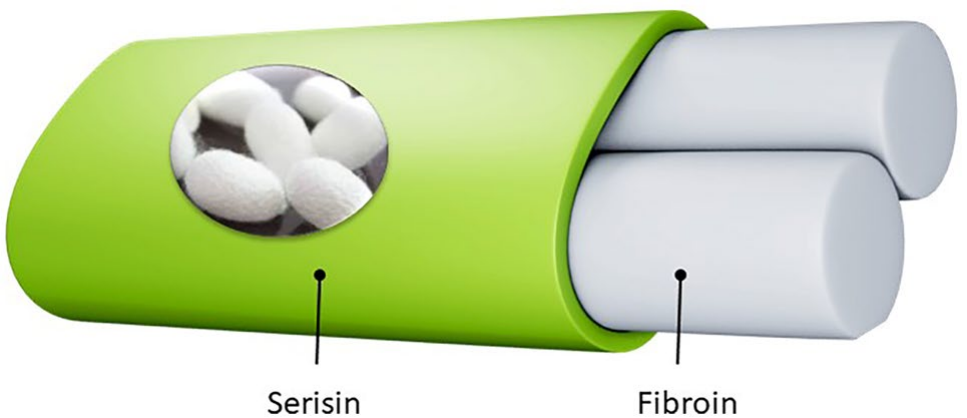
Silk cocoon.

Bombyx mori *mulberry* silk protein fibers are made up of two proteins that help make cocoons: silk sericin (SS), which forms the outer layer, and silk fibroin (SF), located in the core (Fig. 1). During raw silk production, sericin, which binds the silk fibers together within the cocoon, dissolves readily in hot water, facilitating the reeling process^1,2^. The processing of raw silk generates 600,000 tons of waste liquid, from which Kunz et al.^3^ recover 150,000 tons of SS. Silk processing facilities discard tons of SS as waste during raw silk processing, without reusing them^4^. The recovery of SS, which is obtained from a renewable source instead of petroleum and reclaimed from waste, is important as it represents both an environmentally friendly and economically viable resource and can be used as raw material in the production of specialized materials In this study, sericin obtained from Bombyx mori (mulberry) cocoons was used. Bombyx mori sericin, which is mulberry silk, shows more consistent properties than other species thanks to its domesticated structure and controlled growing conditions. In addition, due to the high content of hydrophilic amino acids, it interacts easily with water. Mulberry sericin protein is rich in hydrophilic amino acids, especially serine, threonine, and aspartic acid. This structure supports many biomedical properties of sericin, such as biocompatibility, antioxidant, moisture retention, and wound healing^5,6^.

The SS protein is applicable in various significant industrial sectors, such as chemical, cosmetics, pharmaceuticals, biomedicine, food, and textiles^7–9^. The polar side chains of hydroxyl, carboxyl, and amino groups in SS enable its cross-linking, copolymerization, or blending with other polymers to create advanced biodegradable materials with improved qualities^10,11^. The reactive functional groups on SS make it susceptible to chemical modifications and thus SS offers significant potential for covalent chemical modification^12,13^. Despite this, reports on the chemical modification of SS are still relatively scarce in the literature.

Teramato et al. (2004) investigated the homogeneous modification of silk sericin with 4-cyanophenyl isocyanate in LiCl/DMSO solution. As a result of the modification, significant changes were observed in the solubility, hygroscopic properties and thermal stability of sericin^14^.

In another study, sericin was chemically modified with cyanuric chloride by Pattanaik et al. and this modified sericin was applied to textile surfaces such as wool, silk and polyester. It was determined that the fabrics gained antimicrobial properties and their washing fastness increased after the modification^15^.

Covalent modification, which entails the attachment of diverse chemical groups to a protein through covalent bonds to modify its conformation, is a technique for activating or inhibiting proteins. This enables the targeted alteration of physicochemical parameters for certain objectives^16^. A nucleophilic substitution (SN_2_) process facilitated the replacement of hydroxyl/carboxyl groups in sericin with primary/secondary amine groups^17,18^. The amino groups within the structure are the primary functional groups for chemical modification. Amino groups in their cationic state are especially advantageous due to their ability to provide a permanent positive charge, which is beneficial for characteristics such as water solubility. Based on their reactivity, pKa values, and the acidity of the environment, primary, secondary, and tertiary amino groups can be reprotonated to reinstate the cationic charge. Primary and, in particular, secondary amino groups are appropriate for amine coupling processes. Such reactions typically occur swiftly and with high yield in aqueous conditions^19^.

Nanofiber synthesis is an interesting and effective technique for employing waste proteins like SS^20,21^. Silver sulfide nanofibers are of considerable interest for medicinal applications, regenerative medicine, and other industrial materials. Composite polymeric nanofibers containing silk proteins are advantageous for healthcare applications due to their smooth surfaces, non-aggregation properties, and dense mesh architectures with small pores^22^.

Electrospinning is a multifaceted technique that facilitates the fabrication of diverse materials characterized by exceptionally high surface-to-volume ratios and intricate fiber structures^23^. Electrospun mats are highly beneficial across various sectors. They can be utilized in healthcare^24,25^, cosmetics, food, textiles, and filtration^26,27^ and can be engineered to possess distinctive characteristics such as elevated oxygen permeability, structural resemblance to the extracellular matrix (ECM), extensive surface area, minimal volume, and customizable pore size.

Coaxial electrospinning, a kind of electrospinning, generates multilayer core–shell nanofibers that offer distinct advantages. It facilitates the formation of diverse material combinations. The adjustable core–shell size ratio facilitates the regulation of core–shell fiber attributes, including stack thickness, mechanical reinforcement, electrical and magnetic capabilities, biocompatibility, antibacterial and anticancer qualities, and photovoltaic features. Functional materials such as nanoparticles, nanotubes, pharmaceuticals, proteins, and living cells can be readily integrated into core–shell fibers by dispersing or dissolving them in electrospinning solutions. The coaxial electrospinning technique enables the formation of core–shell fibers composed of two or more distinct polymers within a single fiber. This enables the integration of the advantageous mechanical characteristics of synthetic polymers with the biocompatibility of biomaterials for cellular applications^28,29^.

The SS protein is predominantly composed of the amino acid serine. This research altered SS (M-SS) by transforming the hydroxyl groups of SS amino acids into amino derivatives via reactions with ammonia (NH_3_), methylamine (CH_3_NH_2_), ethylamine (CH_3_CH_2_NH_2_), butylamine (CH_3_(CH_2_)_3_NH_2_), benzylamine (C_6_H_5_CH_2_NH_2_), aniline (C_6_H_5_NH_2_), 1-naphthylamine (C_10_H_7_NH_2_), and furfurylamine (C_5_H_7_NO). Furthermore, we developed a combinatorial multiphase system for SS/M-SS employing suitable core axial methodologies. We fabricated nanofiber composite mats (NCMs) via the coaxial electrospinning technique to improve mechanical strength and biocompatibility. We employed polyvinyl alcohol (PVA) as the carrier polymer and fiber core, while utilizing polylactic acid (PLA) and polycaprolactone (PCL) as the fiber shells. PVA was electrospun as the fiber core to inhibit biodegradability, while PCL and PLA were electrospun as the shell for their capacity to impart requisite mechanical strength and little adherence to injured tissues.

The selection of suitable materials for biomedical applications is of critical importance. Polylactic acid (PLA) and polycaprolactone (PCL) are materials approved by the U.S. Food and Drug Administration (FDA)^30^. However, their hydrophobic nature, slow degradation rate, and low mechanical properties limit their applications in drug loading and release^31^.

Polyvinyl alcohol (PVA) has been selected as a synthetic polymer to enhance the performance of PLA and PCL due to its biocompatibility, hydrophilicity, high mechanical strength, and chemical and thermal stability^32,33^. Additionally, SS is hydrophilic, biocompatible, and biodegradable, with cell adhesion-enhancing properties and high mechanical strength^34,35^.

In this study, considering all these properties, it was aimed to design multilayer nanofibrous structures containing PLA, PCL, PVA and SS/M-SS, where hydrophilic and hydrophobic polymers are combined in a single fiber structure. The biodegradability of these structures, improved thermal and mechanical properties will enable the development of functional materials, especially in the cosmetics, pharmaceutical and biomedical fields.

## Materials and methods

### Materials and devices

Methanesulfonyl chloride, MSCl (≥ 99.7%); poly vinyl alcoho (PVA- mw: 85,000–146,000), polycaprolactone (PCL- mw:70,000–90,000), polylactic acid (PLA- mw ~ 60,000), trifluoroacetic acid (TFA), 25% ammonia solution, naphthylamine, furfurylamine, methylamine solution (40%), triethylamine (TEA), aniline, butylamine, ethylamine, dimethyl sulfoxide, DMSO, chloroform, and lithium chloride (LiCl) were supplied from Merck. The silk sericin protein used in this study was extracted from the cocoons of the mulberry silkworm (Bombyx mori). It was supplied by IDO BIO INC. and appears as a light yellow powder. The molecular weight of the cosmetic-grade sericin derived from silk pupae is approximately 1.5–2 kDa. Sodium dodecyl sulfate (SDS), 3-(4,5-dimethylthiazol-2-yl)-2,5-diphenyltetrazolium bromide (MTT) and phosphate-buffered saline (PBS) were purchased from Sigma Aldrich. The L929 mouse fibroblast cell line was obtained from the American Type Culture Collection (ATCC). Dulbecco’s Modified Eagle Medium (DMEM), fetal bovine serum (FBS), penicillin and streptomycin were supplied from Gibco.

Fourier transform infrared (FTIR) spectra of SS/M-SS products and NCM were obtained using a JASCO 6600 model FTIR spectrometer in the wavelength range of 400–4400 cm^−1^. The chemical structures of amine-modified sericins were examined using proton nuclear magnetic resonance spectroscopy (^1^H-NMR) with a Varian Unity Inova 500 MHz NMR spectrometer. The sample was dissolved in DMSO-d6. Temperature control was maintained between − 80 °C and + 130 °C. The thermal properties of amine-modified sericines were analyzed using Thermogravimetric Analysis (TGA). The TGA characterization was performed on TA Instruments TGA-55 model TGA analyzer in a nitrogen (N_2_) atmosphere, with a heating rate of 10 °C/min over a temperature range of 20–800 °C, and SS/M-SS were weighed at 2 mg. Additionally TGA characterization of SS/M-SS doped nanofiber composites analyzed in a nitrogen (N_2_) atmosphere, with a heating rate of 20 °C/min over a temperature range of 20–650 °C and samples weighing 2 mg. Thermal analysis was also performed using a Hitachi DSC 7000X model differential scanning calorimetry (DSC) analyzer in a nitrogen (N_2_) atmosphere with a heating rate of 10 °C/min over the temperature range of -70 to 220 °C. The tensile tests of nanofiber composites were conducted according to ASTM D882-10 standards using a DEVOTRANS DVT UZM K3 model tensile-compression testing device. Surface characterization of the produced SS/M-SS doped nanofiber mats was carried out using an FEI QUATRO S SEM scanning electron microscope. Cells was continuously monitored using an inverted microscope, Leica DMi1-11090137001.

Nanofiber composites were produced using Inovenso, Nanospinner 24-XP coaxial nozzle electrospinning system.

### Silk sericin modification synthesis

**Fig. 2:**
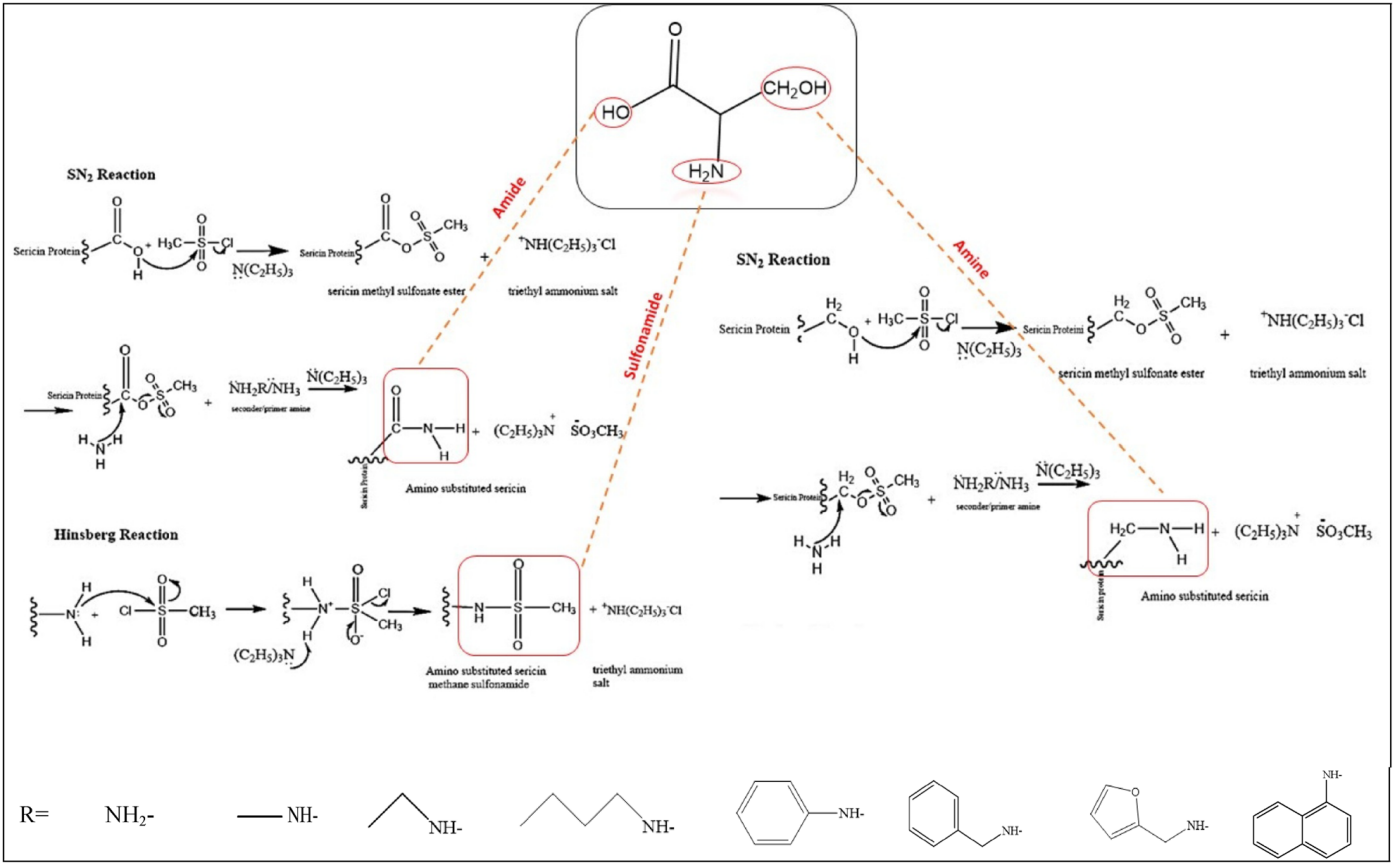
Reaction mechanism of Sericin modification.

The modification of sericin was developed based on the procedures studied by Teramoto^14^, Noossak^36^ and their colleagues. Accordingly, 0.5 g of sericin was stirred in 10 mL of a LiCl/DMSO/1M solution for 3 h (A). Simultaneously, 0.005 mol/0.4 mL MsCl was stirred in 10 mL of a LiCl/DMSO solution for 2 h (B). Subsequently, solution A was added to solution B, and stirring continued overnight. 0.005 mol (0.69 mL) of TEA was gradually added dropwise to the A + B mixture. The stirring was continued for 2 h. 0.005 mol of primary/secondary amine (Table S1) and TEA (0.005 mol/0.69 mL) were added dropwise to this mixture simultaneously (at 60 °C), and the stirring was continued overnight. After the reaction was completed, the mixture was poured into 50 mL of DCM. It was then stored in the refrigerator for 1 h to induce precipitation. The precipitated material (M-SS) was separated by centrifugation at 3000 rpm for 10 min, and the final products were lyophilized at − 80 °C. The synthesis reactions of amino-substituted sericins are depicted in Fig. 2.

The amino groups that replaced the –OH groups in the structure of the sericins obtained from the synthesis, along with their molecular formulas and nomenclature, are presented in Table S1. In each formula, the modification was performed using a single type of amine.

As shown in Fig. 2, all hydroxyl groups were modified into amine-based derivatives. Sulfonamide groups were formed as a result of the Hinsberg reaction between free amino groups in sericin proteins and MsCl^37,38^. In addition, amides were obtained from the free carboxyl groups, alkyl amines from alkyl hydroxyls. Methanesulfonate (MsCl) salts were used, as they are considered good leaving groups in nucleophilic substitution reactions^39^.

### NCM production with coaxial electrospinning technique

Polymer solutions were used to produce the nanofiber composites listed in Table 1 using an electrospinning system with a coaxial nozzle. A dual syringe pump and coaxial nozzle were utilized in the electrospinning system^40–47^.

**Table 1:** Optimized M-SS doped NCM production parameters by electrospinning technique with coaxial nozzle.

Code of NCM with M-SS doped	1. Syringe pump (shell layer) (%)	2. Syringe pump (core layer)
SNF1	7.5 PCL	–
SNF2	7.5 PLA	–
SNF3	10 PVA	–
SNF4	7.5PCL-7.5 PLA	10 PVA
SNF5	7.5 PCL-7.5 PLA	7.5 SS-10 PVA
SNF6	7.5 PCL-7.5 PLA	7.5 SAMT-10 PVA
SNF7	7.5 PCL-7.5 PLA	7.5 SMAT-10 PVA
SNF8	7.5 PCL-7.5 PLA	7.5 SEAT-10 PVA
SNF9	7.5 PCL-7.5 PLA	7.5SBUAT-10 VA
SNF10	7.5 PCL-7.5 PLA	7.5 SANT-10 PVA
SNF11	7.5 PCL-7.5 PLA	7.5 SBAT-10 PVA
SNF12	7.5 PCL-7.5 PLA	7.5 SFUAT-10 PVA
SNF13	7.5 PCL-7.5 PLA	7.5 SNAT-10 PVA

As hydrophobic polymers, PCL and PLA solution mixtures (shell solution) were prepared by stirring in chloroform/DMF (60/40) solvent system at 60 °C for 1 h^48,49^. Using a coaxial nozzle, it was sprayed from the outer nozzle with a syringe pump feed flow of 2 mL/h.

**Fig. 3:**
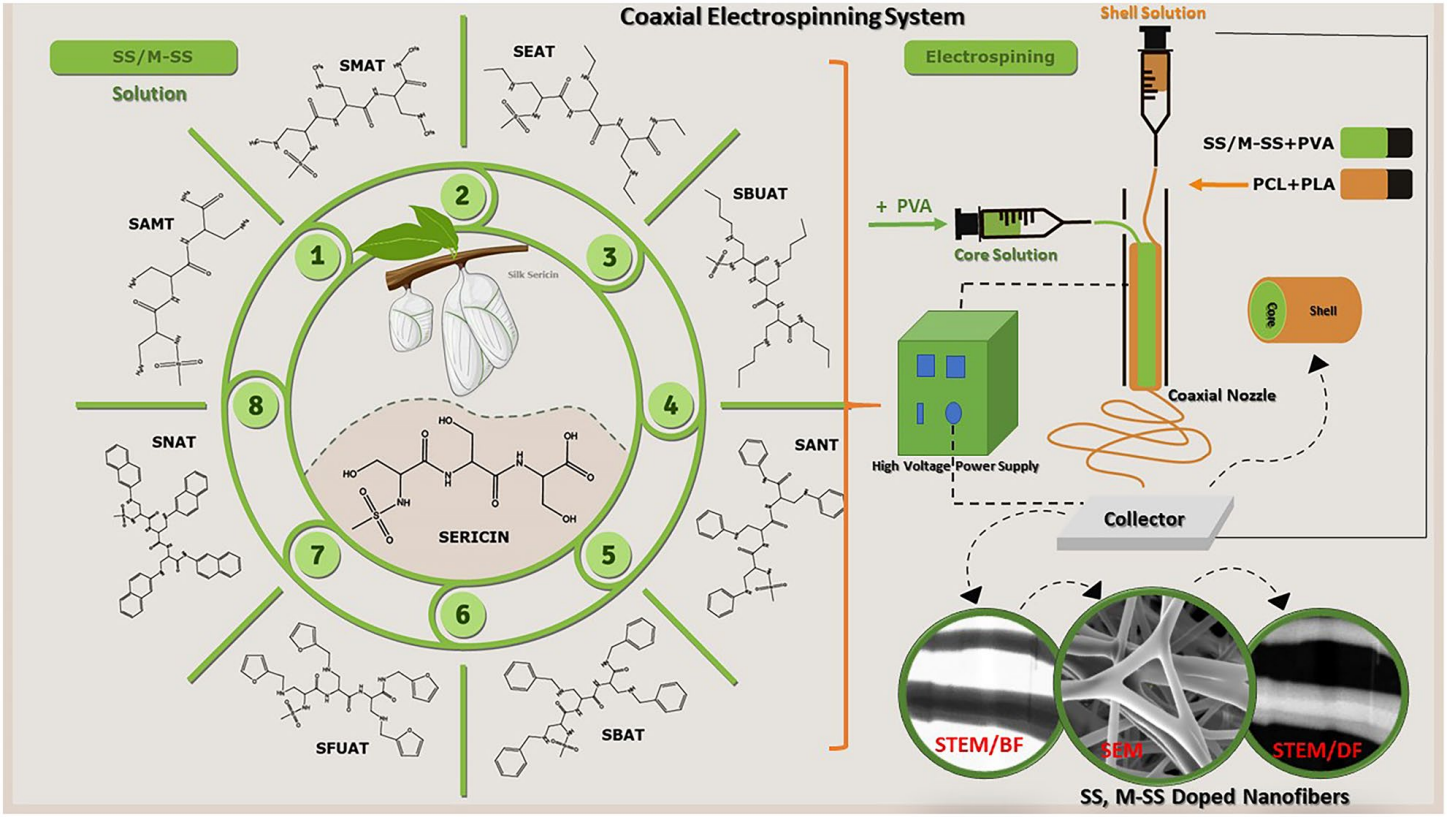
Schematic representation NCMs production with coaxial electrospinning technique.

A hydrophilic polymer, PVA, was prepared by stirring it in a TFA/Distilled Water (50/50) solvent system. SS/M-SS materials were then added (core solution), which was stirred at 55 °C for 1 h. Subsequently, it was sprayed from the inner nozzle using a syringe pump with a feed rate of 2 mL/h^50^. Other working conditions for the electrospinning process were applied in the voltage range of 25–30 kV, with a working distance of 10–15 cm. Before the characterization studies, the produced nanofiber composites were dried for 24 h in a vacuum oven at 50 °C to ensure the removal of solvents from the nanofiber composites. The production stages of the NCMs in the electrospinning process are shown in Fig. 3.

### Cell culture studies

The culture of L929 mouse fibroblast cells (ATCC) was performed using DMEM containing 10% FBS, along with 0.5% penicillin and 0.5% streptomycin, and the coverage of the flask’s base by the cells was continuously monitored using an inverted microscope. Cell lines were cultured in at 37 °C incubator with 5*% *CO_2_ and passaged when they reached approximately 80% confluence. Experiments were performed on cells between passage 5 and 12. Cells at approximately 80% confluence were passaged using 1 ml (10x) trypsin–EDTA, the detached cells collected from the flask and transferred into a 15 mL conical falcon tube. After centrifugation at 1000 rpm for 5 min, the supernatant was discarded and the pellet was resuspended with 1 mL of fresh media to final volume, followed by cell counting. Based on the cell count, 4 × 10^5^ cells were distributed evenly into each T25 flask with 3 mL of DMEM containing 10% FBS, which had been previously added to the flasks. Cell proliferation was observed every 24 h, and when cells reached 80% confluence, it was determined that they were ready for passage, and the passaging process was initiated. In all experiments, fibers were sterilized under UV light for 2 h. They were washed with PBS and subsequently saturated with the culture medium before cell seeding was performed. The results were calculated by comparing them to the control group.

#### MTT assay for in vitro testing cell viability

All fibers were analyzed according to the ISO 10993-5 standard for in vitro biocompatibility testing, and MTT method was performed as previously described by Demir et al.^51^. MTT is a chemical compound used to assess cellular activity in living cells. It is a water-soluble tetrazolium salt, which is converted by the succinate dehydrogenase enzyme in the mitochondria of living cells into an insoluble, purple-colored formazan. Since the nanofiber mat is impermeable to formazan, it accumulates within living cells. L929 cells were seeded into 96-well plates (8 × 10^3^ cells/well) and incubated for 48 h under incubator at 37 °C and 5*% *CO_2_. After incubation, removed the incubation medium and replaced with application medium at varying concentrations (50%, 75%, and 100%) and incubated for 24 h at 37 °C. At the end of the experiment period, the application medium was removed and 110 μL/well of MTT solution (10%, 5.0 mg/mL PBS) was added to the wells. The cells were incubated for 4 h under at 37 °C. Subsequently, 100 μL of SDS (1.0 g SDS, 10 mL PBS, 0.01 M HCl) was added to dissolve the formazan crystals. After 24 h in the incubator at 37 °C, UV–Vis absorbance was measured at 570 nm with a reference at 630 nm using a microplate reader (ThermoFisher, Waltham, MA, USA). All fiber test materials were sterilized and extracted according to ISO 10993-12:2021 standards. For extraction, the test sample was immersed in DMEM at 37 °C for 24 h. After 24 h, the fibers were removed, and the remaining medium was defined as 100% extract application medium. The color of the extract, as well as the presence of any particles or turbidity, was noted before and after extraction. No pH adjustment, filtration, centrifugation, or particle removal methods were applied to the extract used on the cells.

#### Cell fixation, DAPI staining (fluorescent microscopy and SEM imaging)

L929 cells are monolayer cells that actively adhere to the surface for proliferation. DAPI staining, which stains the nuclei of cells, was used to observe whether the cells adhered to the prepared fibers and retained their ability to proliferate. A total of 20 × 10^3^ L929 cells were seeded onto fibers placed in six-well plates, covered with DMEM medium, and incubated for 48 h. After incubation, the medium was removed, and each well was washed three times with PBS. Cells were incubated with DAPI solution for 15 min to stain the nuclei. Finally, the cells were washed three times with PBS, and the images were obtained using a fluorescence microscope (Leica, DMI4000B) with a DAPI filter^52^. For SEM imaging, the cells were fixed with 4% formaldehyde instead of being stained with DAPI, washed three times with PBS, and subsequently coated with gold for SEM imaging (Fig. 13).

## Results and discussion

### Characterization of amine modified sericines (M-SS)

#### FTIR spectroscopy

**Fig. 4:**
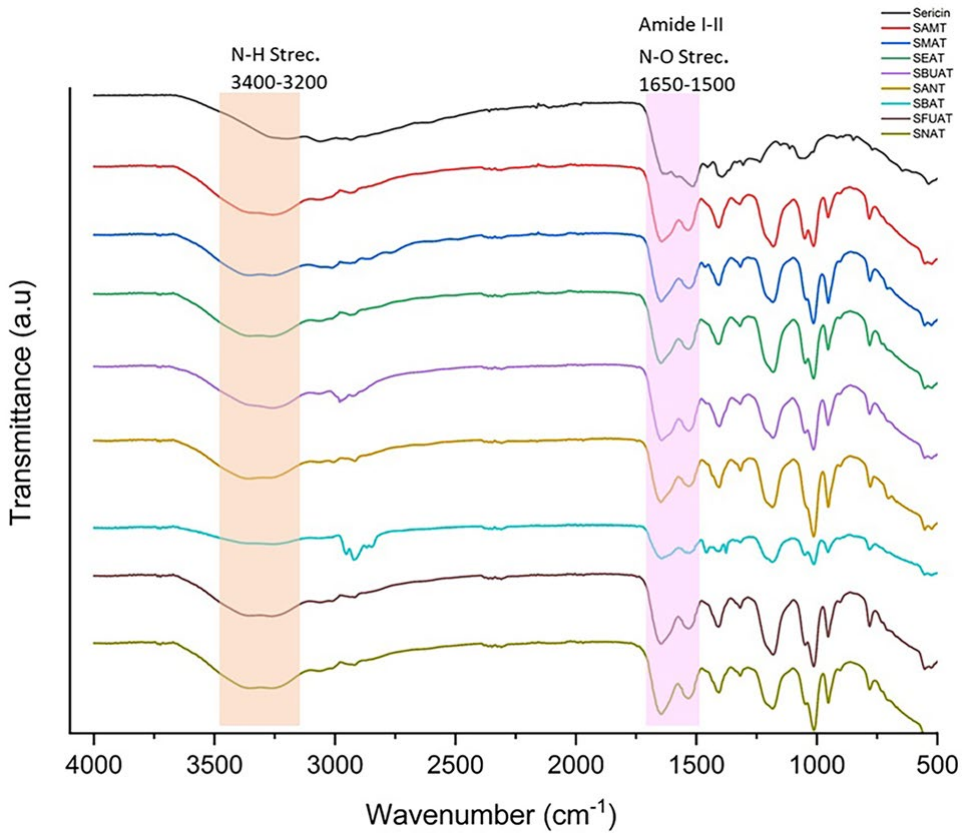
FTIR spectra of amine modified sericines.

The comparative FTIR spectra of SS/M-SS are presented in Fig. 4, and the key peaks observed in these spectra are listed in Table 2. The conversion of spectra and the analysis of SS, M-SS structres were performed using the Origin (OriginLab Corp., USA) software program.

**Table 2:** FTIR spectra peaks of the amine modified sericines.

Sericin derivatives	Amide I (N–H) peek (cm^−1^)	Amide II (N–H) peek (cm^−1^)	Amide I (C–N) peek (cm^−1^)	Amide II C–N peek (cm^−1^)	Others (1400–400) peek (cm^−1^)	References
SAMT	3380	3261	1644	1535	1406, 1318, 1265, 1051	^55^
SMAT	3335	3239	1638	1529	1406, 1315, 1267, 1015	^56^
SEAT	3360	3260	1648	1531	1404, 1316, 1223, 1012	^57^
SBUAT	3343	3267	1647	1505	1407, 1318, 1225, 1014	^58^
SANT	3335	3245–3045	1649	1529	1403, 1315, 1224, 1013	^59^
SBAT	3343	3218–3049	1648	1524	1405, 1314, 1231, 1015	^60^
SFUAT	3355	3255–3063	1644	1538	1404, 1316, 1231, 1012	^61^
SNAT	3386	3262–3060	1645	1534	1405, 1318, 1235, 1012	^62^

The M-SS FTIR spectrum revealed characteristic peaks corresponding to aromatic and aliphatic C–H bonds at 3100–2800 cm^−1^, N–H bonds of amines at 3400–3200 cm^−1^, N–H bonds of amide groups at 3300–3400 cm^−1^, C=N peaks of amide groups at 1650–1500 cm^−1^, and stretching vibration bands of C–N bonds in the 1200–1300 cm^−1^ range^53^. After modification, an increase in the number of amide groups was observed in the SS structure, as confirmed by more intense absorption bands in the 1650–1500 cm^−1^ range, consistent with modified molecular structures (supp. info.Table S1). In the SS structure, the intensity of the amide I band is quite low. However, after the chemical modification of SS using amine reagent, the intensity of the amide I band (1650–1600 cm^−1^) increased in the infrared spectra of all M-SS samples. Similarly, while the N–H stretching bands appear at approximately 3200 cm^−1^ in the FTIR spectrum of SS, the intensity of these bands increases in the 3400–3200 cm^−1^ region in the infrared spectra of M-SS samples. According to the reaction mechanism shown in Fig. 2, the OH groups present in the SS protein structure have been replaced by amino groups.

The peaks between 1500 and 400 cm^−1^ could be associated with complex amide III and/or IV bands^54^. The FTIR spectra of SS/M-SS structures showed intensified peaks in this range. FTIR spectra of modified sericins with aromatic amines (benzylamine, naphthylamine, furfurylamine, aniline) also exhibited aromatic C–H stretching bands in the 3100–3000 cm^−1^ range across all four spectra. Similar observations were also reported by Al-Tabakha et al.^53^. When aliphatic amine groups bind to the SS structure, the NH stretching band in the 3400–3200 cm^−1^ region becomes broader. However, when aromatic amine groups bind to the SS structure, additional aromatic C–H bands appear in the 3000–3100 cm^−1^ region.

#### ^1^H-NMR spectroscopy

**Fig. 5:**
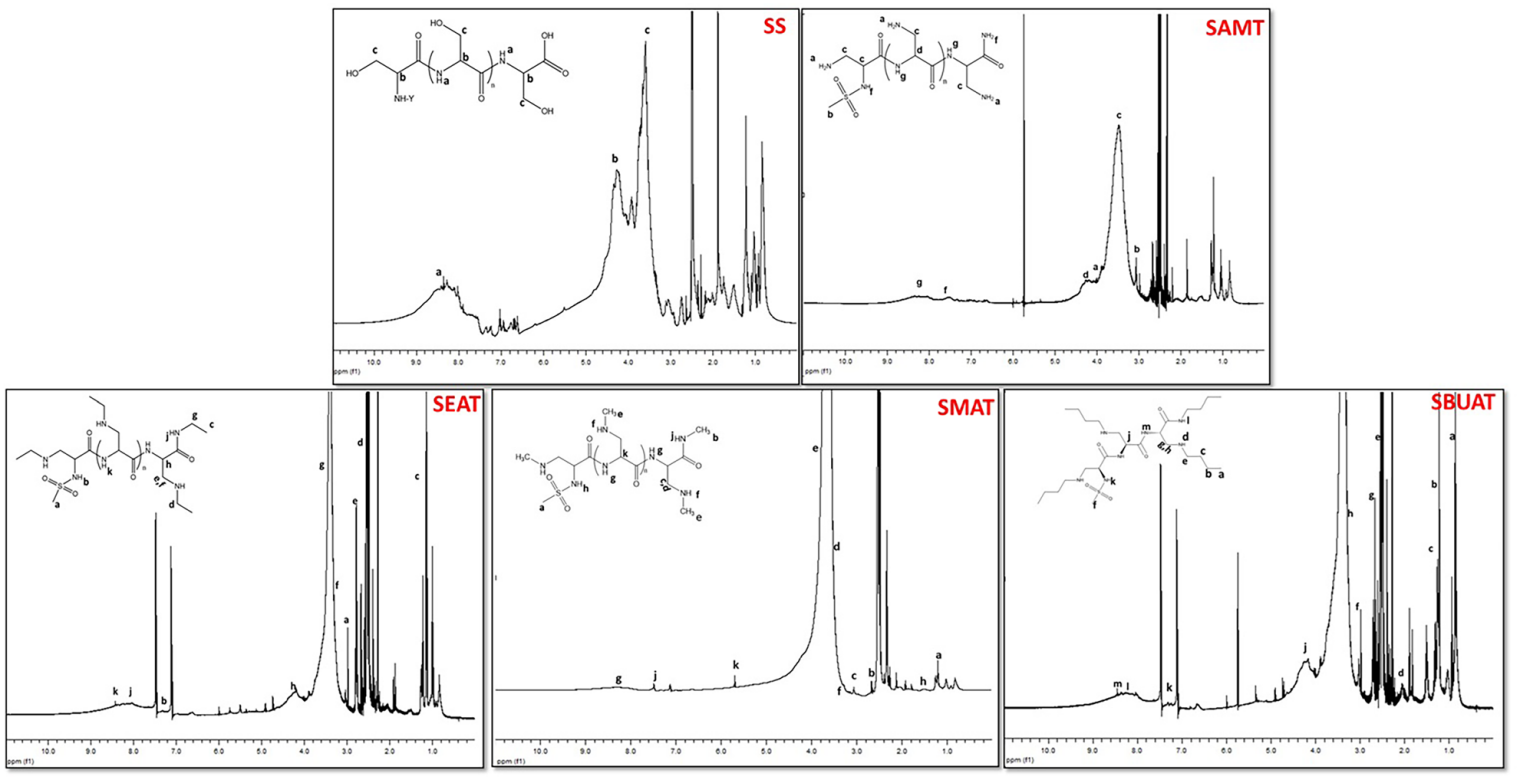
^1^H-NMR Spectra of SS/SS modified with aliphatic amine groups.

**Fig. 6:**
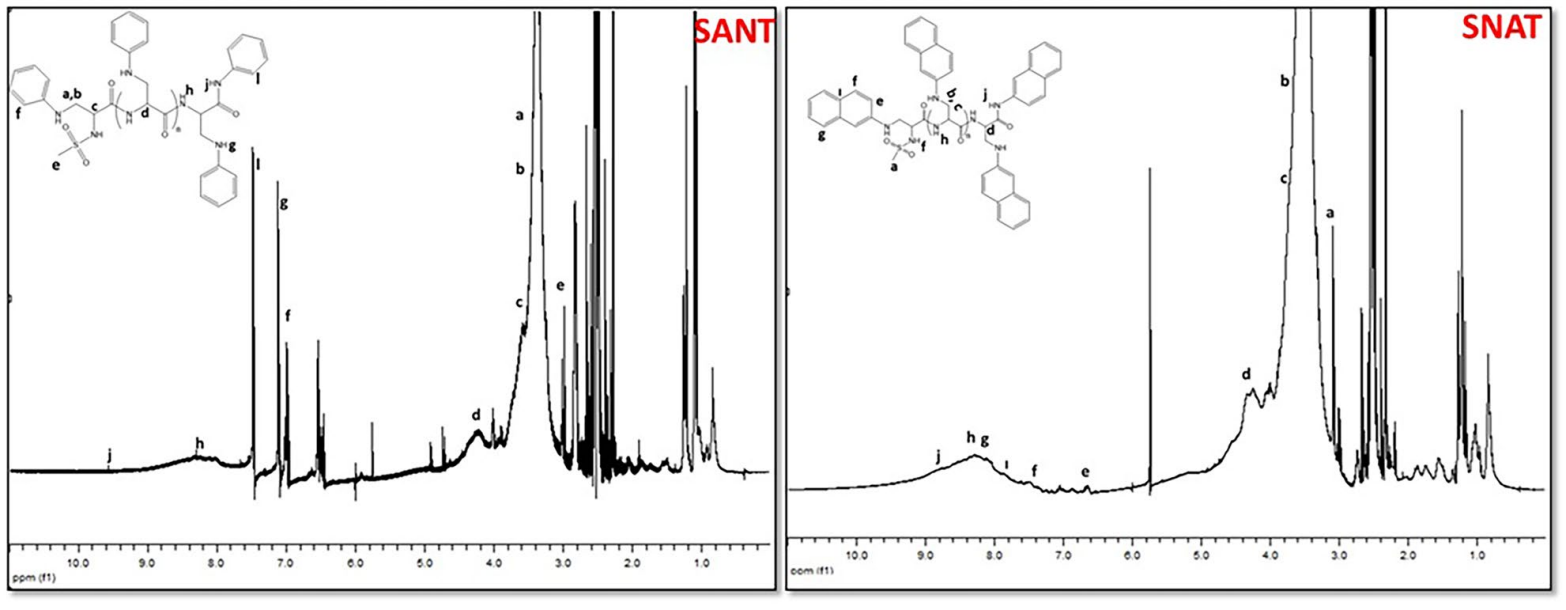
^1^H-NMR Spectra of SS modified with aromatic amine groups.

**Fig. 7:**
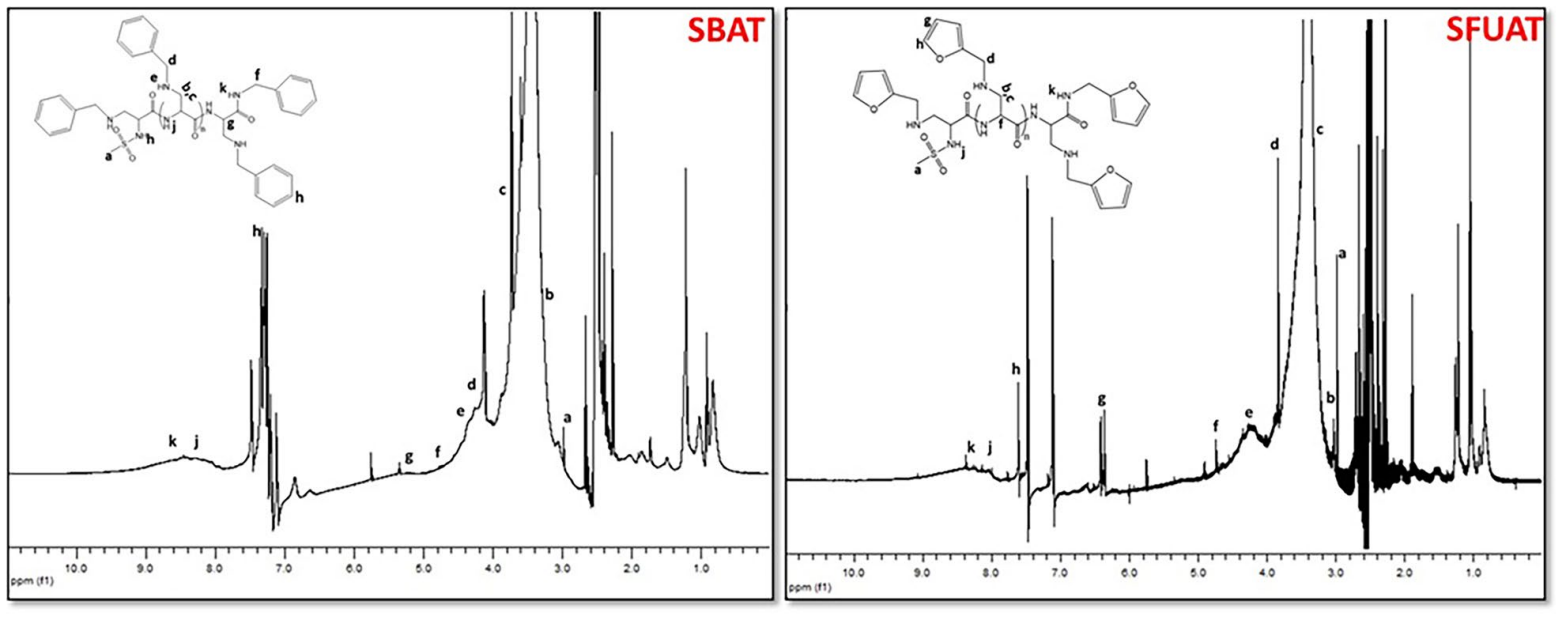
^1^H-NMR Spectra of SS modified with aliphatic–aromatic amine groups.

^1^H-NMR spectra of sericin, sericin modified with aliphatic amines, aromatic amines, and sericin modified with both aromatic and aliphatic amines are given Figs. 5, 6 and 7. Samples were dissolved in DMSO-d₆, and the residual solvent signal appeared as a singlet at 2–2.50 ppm.

In the ^1^H-NMR spectrum of sericin, three main peaks were observed for the sample prepared in D-DMSO solution. Peaks originating from the amide hydrogens (H) of the sericin protein appear around 8–9 ppm. The hydrogen attached to the carbon bound to a nitrogen atom (CH–N) provides another characteristic peak, observed at approximately 4.6 ppm. Another characteristic peak comes from the hydrogens attached to –CH_2_ groups bonded to -OH groups, which appear at around 3.8 ppm. Normally, if sericin consisted solely of serine amino acids, no other peaks would be expected; however, peaks between 1 and 3 ppm are present, likely originating from aliphatic hydrogens of different amino acids in the sericin group. The methyl groups of amino acids such as valine, isoleucine, and threonine give rise to peaks between 0.5 and 1 ppm. Other amino acids are also present in this range^63–65^.

SAMT modification, the ^1^H-NMR spectrum shows that the peak for the protons in the amide group formed in place of the carboxylic acid group (–CONH) appears between 8–9 ppm, while the protons of the –NHCOCH– group are around 4.5 ppm. The peak for the proton in the S–NH group of the protein appears between 7–8 ppm, and the sulfonamide methyl group (–O_2_S–CH_3_) shows a sharp single peak at 2.9–3.0 ppm. The –NCH group in the sulfonamide structure resonates between 3 and 3.5 ppm. Protons of the H_2_NCH- group formed by the addition of the ammonia group to the SAMT structure resonate between 3 and 3.5 ppm, and the RNH protons of the amides appear between 3 and 4.5 ppm (Baker-Tripp, 2018). The –CH protons attached to the amide group resonate further downfield compared to alkyl group protons due to the electronegativity effect of the amide group, which decreases the electron density around the proton^66^.

In the ^1^H-NMR spectrum of SMAT modification, the peak for the protons in the amide group (–CONH) appears between 8–9 ppm, while the protons of the –NHCOCH– group resonate around 5.5 ppm. The S–NH proton peak is between 7 and 8 ppm, and the sulfonamide methyl group (–O_2_S–CH_3_) shows a sharp peak at 2.9–3.0 ppm. The HNCH3– group protons in SMAT resonate between 3 and 3.5 ppm, and the CHNH protons of the amides appear between 3 and 4.5 ppm^67^.

In the ^1^H-NMR spectrum of SEAT modification, the –CONH proton peaks are located between 8–9 ppm, and the –NHCOCH– group protons appear around 4.5–5.0 ppm. The S–NH proton peaks are between 7–8 ppm, and the sulfonamide methyl group (–O_2_S–CH_3_) exhibits a sharp peak at 2.9–3.0 ppm. The HNCH_2_CH_3_– group protons resonate between 2.5 and 3.5 ppm, and the HNCH_2_CH_3_ proton resonates between 1 and 1.5 ppm. The CHNH protons of the amides resonate between 3 and 4.5 ppm^68^.

SBUAT modification, the peaks for the –CONH protons are between 8 and 9 ppm, while the –NHCOCH- group protons resonate around 4.5–5.0 ppm. The CHNH protons of the amides resonate between 3 and 4.5 ppm. The S–NH proton peaks are between 7 and 8 ppm, and the sulfonamide methyl group (–O_2_S–CH_3_) shows a sharp peak at 2.9–3.0 ppm. Protons from the butyl group attached to the amine HNCH_2_CH_2_ CH_2_CH_3_ in SBUAT resonate between 2.5 and 3.5 ppm, while the HNCH_2_CH_2_CH_2_CH_3_ proton resonates between 1 and 1.5 ppm, and the HNCH_2_CH_2_CH_2_CH_3_ proton resonates around 1.0 ppm^69^.

SANT modification shows that the –CONH proton peaks are located between 8 and 9 ppm, and the –NHCOCH– group protons appear around 4.5 ppm. The S–NH proton peaks are between 7 and 8 ppm, and the sulfonamide methyl group ((–O_2_S–CH_3_) shows a sharp peak at 2.9–3.0 ppm. The –NCH group resonates between 3 and 3.5 ppm, and the aromatic CH protons of the aniline group resonate between 6.5 and 7.5 ppm (Das 2020). The NH proton of the amide group, located between an aromatic group and a carbonyl group, experiences a downfield shift due to the electron-withdrawing effects of both groups, causing it to resonate between 9.5 and 10.0 ppm^70^.

SBAT modification, the –CONH proton peaks appear between 8–9 ppm, and the –NHCOCH– group protons resonate around 4.5–5.0 ppm. The S–NH proton peaks are between 7 and 7.5 ppm, and the sulfonamide methyl group (–O_2_S–CH_3_) exhibits a sharp peak at 2.9–3.0 ppm. The –NCH group resonates between 3 nd 3.5 ppm, and the aromatic CH protons of the benzyl group resonate between 7.0 and 7.5 ppm. The –CH proton attached to the benzene ring resonates around 4.0 ppm, and the NH proton of the amide group, situated between two electron-withdrawing groups, resonates between 8.5 and 9.0 ppm^71^.

SFUAT modification, according to ^1^H-NMR spectrum, the -CONH proton peaks are between 8–9 ppm, and the –NHCOCH– group protons resonate around 4.5–5.0 ppm. The S–NH proton peaks are between 7 and 7.5 ppm, and the sulfonamide methyl group (–O_2_S–CH_3_) shows a sharp peak at 2.9–3.0 ppm. The –NCH group resonates between 3 and 3.5 ppm, and the aromatic CH protons of the furfuryl group resonate between 6.5 and 8.0 ppm. The –CH proton attached to the furan ring resonates around 3.5–4.0 ppm^72^.

SNAT modification, according to ^1^H-NMR spectrum, the –CONH proton peaks are between 8.5 and 9.5 ppm, and the –NHCOCH– group protons resonate around 4.5–5.0 ppm. The S–NH proton peaks are between 7 and 8 ppm, and the sulfonamide methyl group (–O_2_S–CH_3_) shows a sharp peak at 2.9–3.0 ppm. The –NCH group resonates between 3 and 3.5 ppm, and the aromatic CH protons of the naphthalene group resonate between 6.5 and 8.5 ppm (Aldilla et al. 2022). Similar to the aniline group, the NH proton in the amide group is located between an aromatic group and a carbonyl group, causing a significant downfield shift, resulting in resonance around 9 ppm^73^.

#### Thermal characterization of amine modified sericines, TGA

**Fig. 8:**
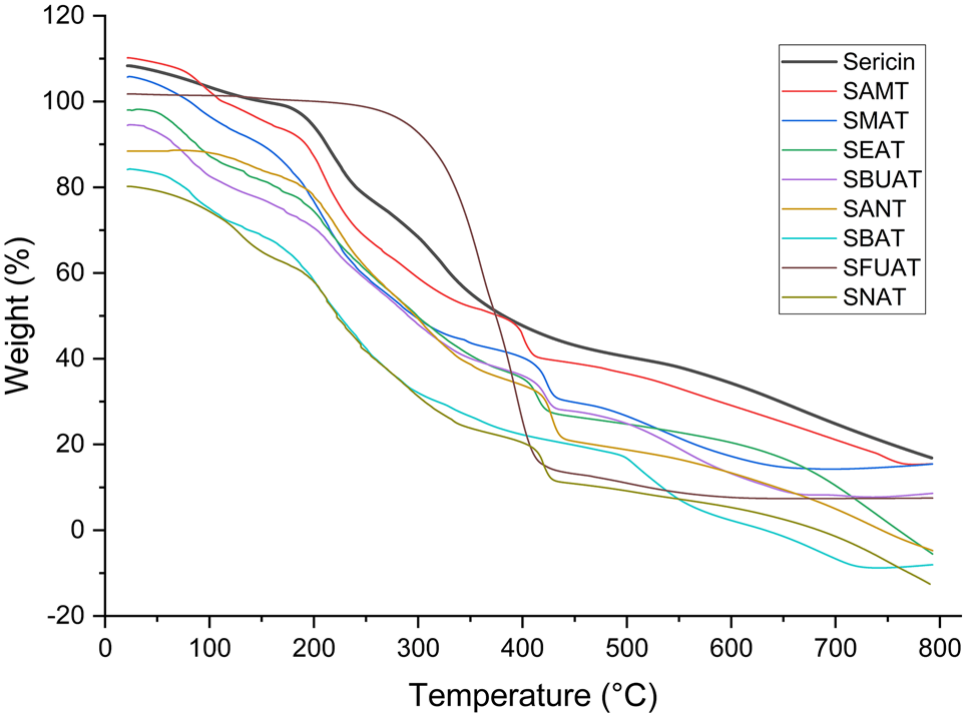
Thermogravimetric analysis (TGA) thermograms of sericin and amine modified sericines.

The TGA thermogram of SS and M-SS products (Fig. 8, Supp. info/Fig. S1) revealed four distinct stages of thermal degradation, corresponding to four mass loss regions. In decomposition temperatures, the onset and end-point minimum temperatures of decomposition were taken as the basis. The temperature ranges for these degradations are provided in Table 3.

**Table 3:** TGA thermograms, decomposition temperature (°C) ranges and weight losses (%) of sericin derivatives.

Sericin Derivatives	Temperature ranges of segmental losses (°C) and weight losses (%)				
	T_1_ (°C)	T_2_ (°C)	T_3_ (°C)	T_4_ (°C)	Reference
SS	50–115 (%10)	115–250 (%24)	250–460 (%35)	460–800 (%23)	^75^
SAMT	50–115 (%10)	115–350 (%47)	350–450 (%12)	450–800 (%20)	^76^
SMAT	50–115 (%10)	115–350 (%50)	350–480 (%15)	480–800 (%14)	^77^
SEAT	45–110 (%10)	120–360 (%46)	360–450 (%12)	450–800 (%27)	^78^
SBUAT	50–120 (%10)	120–350 (%40)	350–450 (%13)	450–800 (%20)	
SANT	60–120 (%10)	120–355 (%40)	355–470 (%17)	470–800 (%21)	
SBAT	50–120 (%12)	120–460 (%52)	460–600 (%17)	600–800 (%11)	^79^
SFUAT	55–125 (%15)	125–370 (%45)	370–570 (%19)	570–800 (%27)	
SNAT	50–150 (%15)	150–350 (%42)	350–460 (%13)	460–800 (%22)	

The thermogram of sericin and amine modified sericines (Fig. 8) shows four distinct mass loss regions representing a staged thermal degradation (supp. info/Fig. S3). According to the TGA thermogram, the first mass loss stage of thermal degradation occurs in the temperature range of 50–150 °C, with a 10–15% mass loss. This stage is associated with the evaporation of water and the dehydration of the samples. The second mass loss stage occurs between 115 and 460 °C, with a mass loss of 25–52%, and is related to the decomposition of volatile compounds and breakdown of amino acid residues in the side chains^74^. Third mass loss stage (T_3_) temperature occurs between 200 and 600 °C, is linked to the degradation of protein components in the molecular structure, such as deamination, decarboxylation, and depolymerization due to the cleavage of peptide bonds. Fourth mass loss stage (T_4_) temperature, representing the final phase of thermal decomposition, occurs between 450 and 800 °C and is associated with the carbonization of the structure. When the TGA thermograms of SS and MSS were examined, an increase in the decomposition temperatures of M-SS was observed, as shown in Table 3. All of the derivatized sericines have higher T_2_, T_3_ and T_4_ values than the reference SS. This indicated that the modifications increase the thermal stability of the sericin. In particular, SMAT, SBAT and SFUAT stand out as the most stable polymers with both higher temperature degradation and wider temperature ranges.

### Characterization of M-SS doped nanofiber composite mats

#### FTIR spectroscopy

**Fig. 9:**
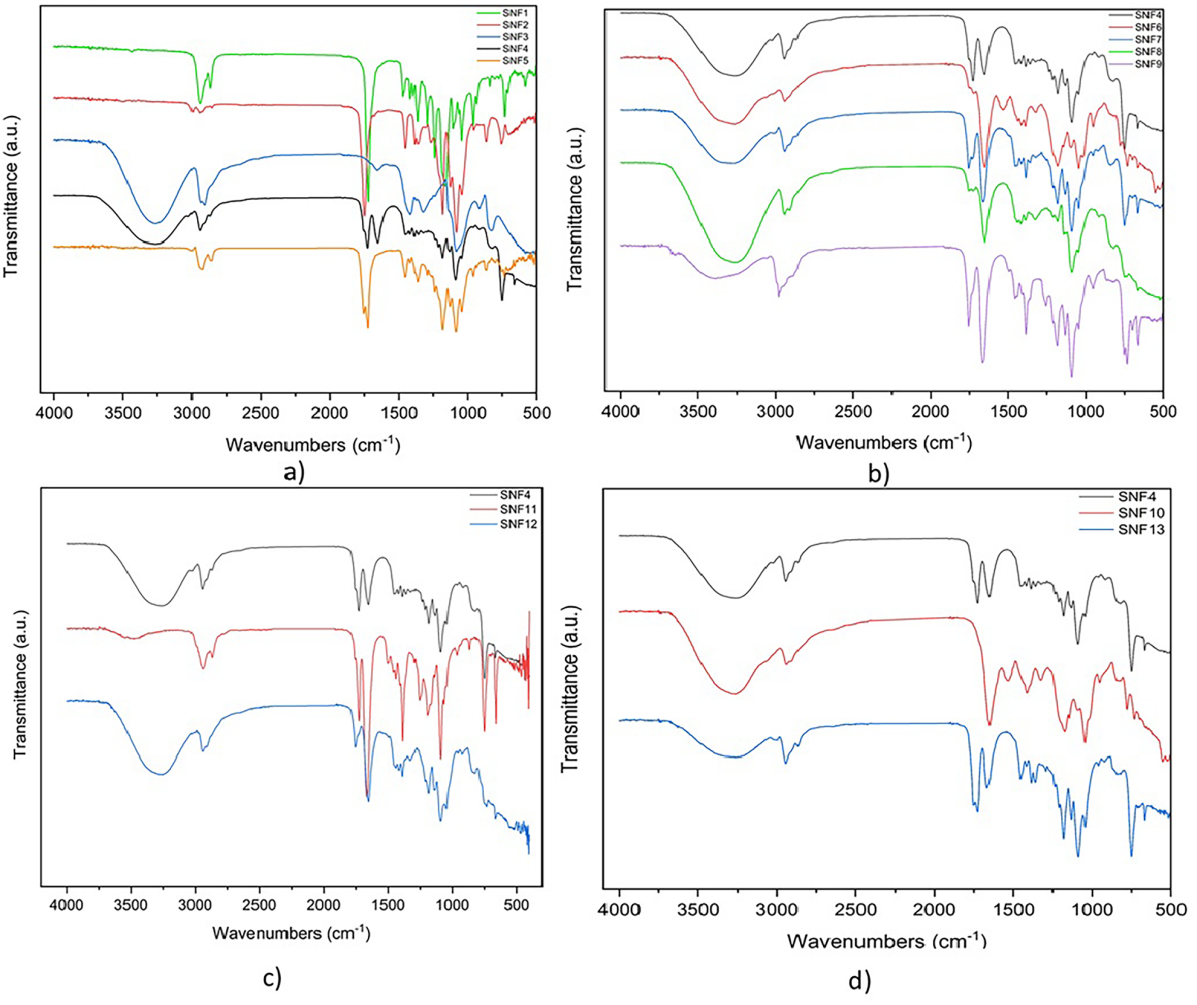
FTIR spectrums of (**a**) the primary matrix polymers, (**b**) M-SS doped nanofiber composites with aliphatic amines, (**c**) M-SS doped nanofiber composites with both aromatic and aliphatic amine groups, (**d**) M-SS doped nanofibers with aromatic amines.

For the structural characterization of NCMs, the FTIR spectra of electrospun mats of each polymer forming the main matrix (SNF1, SNF2, SNF3) were evaluated separately. The FTIR spectra of the main matrix SNF4 (7.5% PCL, 7.5% PLA, 10% PVA) and SNF5 were also examined as shown in Fig. 9a. Furthermore, the FTIR spectra of the mats obtained by incorporating M-SS (SNF6, SNF7, SNF8, SNF9, SNF10, SNF11, SNF12, SNF13) were compared with that of SNF4. It was determined that these spectra were consistent with the SS/M-SS FTIR spectra (See Table 2).

7.5 PCL (SNF1), important bands observed in the FTIR spectrum include the absorption band at 2943 cm^−1^ for C–H asymmetric stretching, and the band at 2863 cm^−1^ for C–H symmetric stretching. The sharp absorption band at 1720 cm^−1^ corresponds to the C=O stretching frequency of the ester carbonyl group. The absorption band at 1239 cm^−1^ is attributed to C–O–C asymmetric stretching, and the sharp band at 1162 cm^−1^ is due to C–O–C symmetric stretching. Thus, the chemical structure of PCL was confirmed by FTIR^80,81^.

7.5 PLA (SNF2), significant bands in the FTIR spectrum include the strong band at 1749 cm^−1^ corresponding to C=O stretching, the bands at 2995 cm^−1^ and 2944 cm^−1^ for CH_3_ C–H stretching, and the band at 1181 cm^−1^ for ester C–O stretching. The FTIR spectrum of PLA is consistent with reported IR spectra in the literature^82^.

10 PVA (SNF3), the FTIR spectrum shows a broad band at 3262 cm^−1^ assigned to hydroxyl groups, the bands at 2937 cm^−1^ and 2908 cm^−1^ for CH_2_ asymmetric and symmetric stretching, respectively. The band at 1416 cm^−1^ corresponds to CH_2_ bending vibrations, the band at 1324 cm^−1^ is attributed to –OH and –CH groups, and the band at 1083 cm^−1^ represents C–O stretching and OH bending vibrations. The FTIR spectrum of PVA is consistent with reported IR spectra^83^.

FTIR spectra of SS doped nanofiber composite mats, SNF5, compared to matrix polymer SNF4; It is clearly seen that the FTIR spectra of SNF5 are significantly changed when compared with the matrix polymer SNF4. According to FTIR specra of SFN5, a broad and weak band appeared at 2996 wavelength for C–H stretching. Also, the signal intensity in this region increased at 2924, 2858 cm^−1^ compared to SNF4. The band at 1453 cm^−1^ corresponds to C–H bending vibrations, while the band at 1364 cm^−1^ corresponds to stretching vibrations of O–H, C–H, and amide III–IV groups. As seen in the spectrum, the signal intensity increased at 1364 cm^−1^, which may corresponds to t amide III–IV groups stretching bands. The sharp absorption band at 1724 cm^−1^ corresponds to C=O from the PCL structure and the strong band at 1754 cm^−1^ corresponds to C=O from the PLA structure.

FTIR spectra of nanofiber composite mats with M-SS additions were analyzed in three groups: (1) M-SS doped nanofiber composites with aliphatic amines, which is depicted in Fig. 9b, (SNF6, SNF7, SNF8, SNF9), (2) M-SS doped nanofiber composites with both aromatic and aliphatic amine groups, which is shown in Fig. 9c, (SNF11, SNF12) and (3). M-SS doped nanofibers with aromatic amines, which is exhibited in Fig. 9d, (SNF10, SNF13). Common peaks identified include C=N amid group peaks in the range of 1650–1500 cm^−1^ and C–N bond stretching vibrations in the range of 1200–1300 cm^−1^. The spectra of nanofiber composites containing aromatic groups (2nd and 3rd groups) also show aromatic C–H stretching bands in the range of 3100–3000 cm^−1^.

#### DSC/TGA

DSC was utilized to study the thermal response of NCMs and to determined their glass transition temperature (T_g_), melting temperature (T_m_), crystallization temperature (T_c_), and melting enthalpy (ΔH).

In the DSC graph, Tc is observed as an exothermic peak due to the crystallization of amorphous regions, while Tm and Tg are observed as endothermic peak, corresponding to melting and increased molecular mobility, respectively (Supp. info/Fig. S2)^84^.

Differences in melting and crystallization temperatures between fibers containing SS and those containing M-SS were noted. Nanofibers with aliphatic amine groups were represented in green, those with aliphatic/aromatic M-SS were in yellow, and those with aromatic amine groups were in gray.

According to Table 4, the nanocomposite mat with the highest crystallization temperature was SNF7^85,86^. DSC graphs are provided in Supp. info/Fig. S2. The melting peaks for PVA, PCL, and PLA nanofiber mats were found to be at 104.39 °C, 217.30 °C, 59.80 °C, and 150.49 °C, respectively^87^.

**Table 4:**
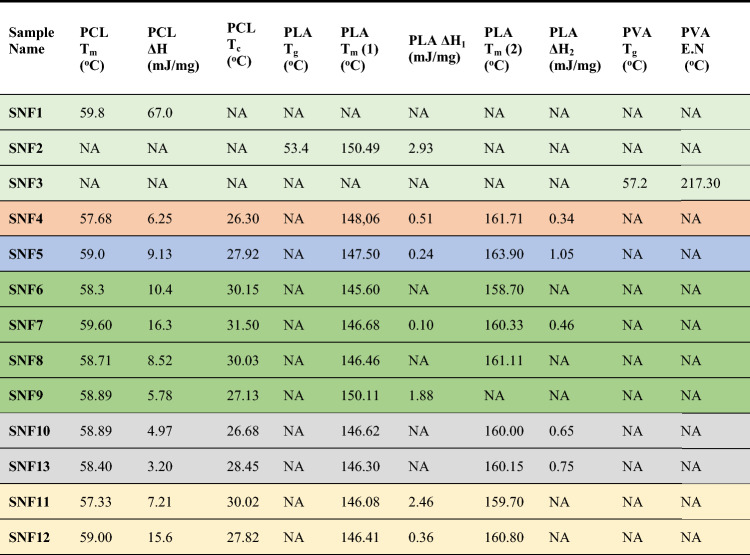
DSC analysis results of M-SS doped NCMs.

The melting enthalpy, melting and crystallization temperature of SFN4, which is the matrix NCM, were analyzed as 6.25 mJ/mg, 57.68 °C, 26.30 oC, respectively. In SNF5 obtained with SS additive, these values were determined as 9.13 mJ/mg, 59.0 °C, 27.92 °C, respectively.

Samples from SNF6 to SNF9 are alkyl substituted amine doped NCMs, and in their DSCs, the maximum heat of fusion (ΔH) is observed as 16.3 mJ/mg in SNF7, 8.52 mJ/mg in SNF8, and this value decreases to 5.78 mJ/mg towards SNF9. According to this result, when the number of carbon atoms in alkyl groups increases, the value of the melting enthalpy decreases. Similar thermal behavior is also observed in crystallization temperatures. Again, the highest Tc value is observed at 31.50 °C in SNF7, which is a methyl amine substituted polymer^88^. This value decreases to 27.13 °C in SNF9, which is a butyl amine doped polymer. SNF10–SNF13 are NCMs with only aromatic substituted amines and their melting enthalpies were measured higher than SNF10 containing benzene group (4.97 mJ/mg), and SNF13 containing naphthyl group (3.20 mJ/mg). The crystallization temperature was 28.45 °C for SNF13 while this value decreased to 26.68 °C for SNF10. SNF11–SNF12 are NCMs with both alkyl and aromatic substituted amines. SNF11 has benzyl amine and SNF12 has furfuryl amine substituents. Higher melting enthalpies were observed in SFN12 (15.6 mJ/mg) and in SNF11 this value was analyzed as 7.21 mJ/mg. The crystallization temperature of SNF11 decreased from 30.02 to 27.82 °C in SNF12.

As a result of comparing these values, it was observed that thermal stability increased with the addition of SS and M-SS.

**Fig. 10:**
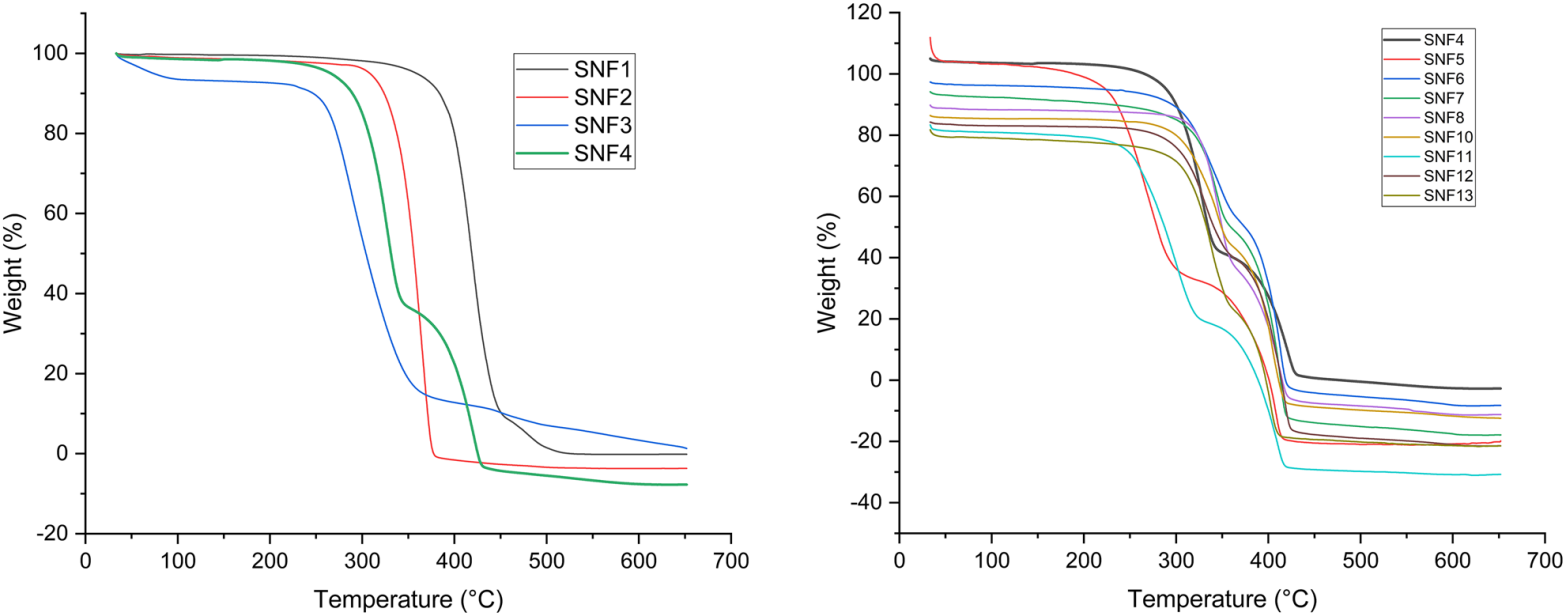
TGA thermograms of SS/M-SS doped nanocomposite mats.

The thermal degradation behavior of M-SS doped NCMs (Table 5) was examined. The thermogram shows curves for temperature (°C) and mass loss (%). Thermograms for SNF1-SNF13 composite mats are shown in Fig. 10. In the TGA thermograms, four mass loss stages are observed (60–366 °C) with specific degradation ranges were identified for each polymer: PCL, 293–448 °C^89^, PLA, 269–383 °C^90^ and PVA, 206–519 °C^91^.

**Table 5:** Decomposition temperature ranges of M-SS doped NCMs on TGA thermogram.

M-SS doped NCM	T_1_ temperature (°C)	T_2_ temperature (°C)
SNF1	293–448	–
SNF2	269–383	–
SNF3	206–519	–
SNF4	220–340	340–430
SNF5	185–320	320–460
SNF6	243–365	365–435
SNF7	275–365	365–435
SNF8	265–365	365–435
SNF9	270–365	365–480
SNF10	250–365	365–435
SNF11	220–335	335–425
SNF12	235–370	370–425
SNF13	260–370	370–425

These polymers each have a single mass loss range. According to the literature, the TGA behavior of these polymers is generally observed to be mass losses due to the dehydration of functional groups in the first step, and the mass losses that occur later are due to the presence of functional groups that are broken off from the main polymer chain. The studies conducted by Ray and Cooney^92^ on this subject reveal similar results. TGA thermograms of M-SS doped mats show two mass loss regions (Table 5).

The thermogram of the SNF4 composite mat (Fig. 10) shows two distinct mass loss regions representing a staged thermal degradation (supp. info/Fig. S3). The first mass loss stage (T_1_) occurs between 220 and 340 °C, with a maximum mass loss of 60%. The second mass loss stage (T_2_) occurs between 340 and 430 °C, with 35% mass loss. The PCL/PLA/PVA nanofiber composite mats show similar thermal characteristics. TGA curves of PVA/PLA/PCL nanofibers exhibit initial sharp declines consistent with the main characteristics of PLA and PVA, followed by gradual degradation. These TGA results indicate that the PCL/PLA/PVA composites were successfully produced by the electrospinning technique. A decrease in the degradation temperatures of PLA/PCL/PVA composite nanofiber mats was observed^93^.

The first mass loss is probably due to dehydration of intramolecular functional groups. The second mass loss between 340 and 430 °C generally occurs due to the separation of functional groups from the main chain. As a result, carbonization and degradation of the polymer main chain occur^94^.

The TGA thermogram of the SNF5 composite mat (Fig. 10) shows two stages of mass loss. Moisture loss occurs between 20 and 185 °C. The first mass loss stage (T_1_) is observed between 185 and 320 °C with a 65% mass loss. The second mass loss stage (T_2_) is between 320 and 460 °C with a 35% mass loss. The addition of SS to the SNF4 matrix resulted in a decrease in the initial degradation temperature.

TGA thermograms of M-SS doped nanofiber composite mats (SNF6–SNF13) show two segmental mass losses. Degradation temperatures are provided in Table 5. The thermal degradation temperatures of modified sericin-doped NCMs (SNF6–SNF13) are higher compared to those of raw sericin-doped NCMs (SNF5). The highest initial degradation temperatures were observed in NCMs SNF7 and SNF9, respectively.

#### Mechanical behavior of M-SS added NCMs in tensile testing

The mechanical properties of SNF1–SNF13 NCMs were determined by applying a 500 N load at a stretching rate of 5 mm/min with a 10 mm jaw gap.

**Fig. 11:**
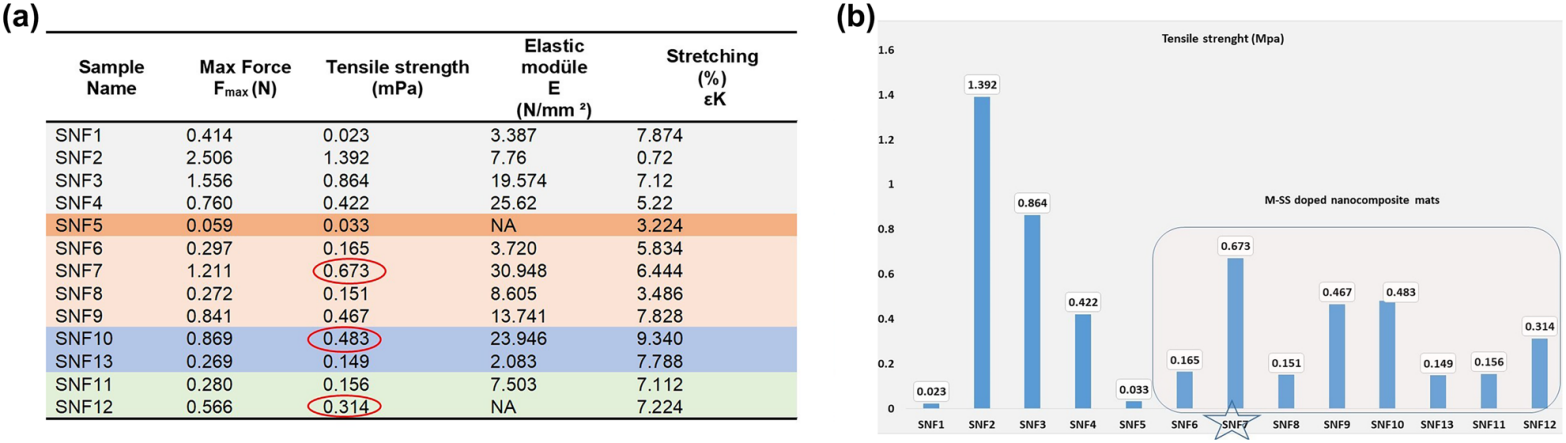
Mechanical behaviour of (**a**) M-SS added NCM in the tensile test result and (**b**) max tensile strength (MPa) chart.

Based on the mechanical analysis results (Fig. 11), SS and M-SS doped NCMs were evaluated in relation to each other. Additionally, the stress–strain curve, which is a fundamental graph of their mechanical behavior, is also presented in supp. info/Fig. S4. This curve shows how much the material stretches (strain) under applied stress.

SNF4 and SNF5 were compared, a significant decrease in tensile strength was observed between SNF4 and SNF5, with values dropping from 0.422 to 0.033 MPa (Fig. 11a), indicating that the substituent introduced in SNF5 adversely affected the mechanical integrity of the NCM. In contrast, the incorporation of M-SS additives appeared to enhance the tensile performance of the materials. Notably, SNF7 exhibited the highest tensile strength at 0.673 MPa, followed by SNF10 and SNF9 with values of 0.483 MPa and 0.467 MPa, respectively. These results suggest that the M-SS group contributes positively to the mechanical reinforcement of the NCM matrix. SNF4 elastic modulus was measured as 25.62 N/mm^2^. However, among all M-SS dopped NCMs, only SNF7 was higher than SNF4 at 30.94 N/mm^2^. In terms of stretchability, an overall increase was observed in the M-SS doped NCMs when compared to SNF4, indicating that M-SS not only improves tensile strength but also enhances the flexibility of the material (supp. info. Fig. S4).

The mechanical behavior of M-SS doped NCMs was analyzed with respect to the three types of substituent group added;1) alkyl substituted amine doped NCMs, SNF6-SN9, 2) only aromatic groups amine doped NCMs, SNF10 and SNF13, and 3) Both aromatic and aliphatic groups amine doped NCMs, SNF11 and SNF12. The tensile strength and elongation properties of these groups were compared. M-SS doped NCMs, (SNF6-SNF13), exhibited a significant increase in tensile strength compared to SNF5, demonstrating nearly 20 times higher tensile strength than only sericin doped nanofiber mats. It was determined that sericin modification increased the tensile strength value in nano composite mats.

Among alkyl substituted amine doped NCMs, SNF7 showed the highest tensile strength at 0.673 MPa and an elongation value of 6.44%. Among the only aromatic groups amine doped NCMs, the tensile strength of SNF10 was superior, with values of 0.483 MPa and 9.34% elongation. Among both aromatic and aliphatic groups amine doped NCMs groups, SNF12, exhibited better mechanical properties compared to SNF11, with tensile strength of 0.566 MPa and an elongation value of 7.22%. Furfurylamine contains a furan ring that is planar, polar, and semiaromatic. This structure allows for improved intermolecular interactions. Compared to the benzene ring, the furan ring is a less conjugated and less rigid aromatic system. This structural difference may have provided some flexibility, reducing the brittleness of the polymer. Strong hydrogen bonds can form between the hydrogen atoms in the furan ring (C–H) and the carbonyl groups (C=O). It can form hydrogen bonds with the hydroxyl and carboxyl groups in the nanofibers. This is thought to improve mechanical integrity by increasing interfacial adhesion and contributing to crosslinking^95,96^.

The nanofiber mat with the highest tensile strength among the three groups was identified as SNF7^97,98^. It was observed that the modifications resulted in higher tensile strength and elongation values in NCMs compared to SS-containing nanofiber mats.

#### Morphological characterization of M-SS doped NCMs by SEM

Morphological analysis by scanning electron microscopy included examination of nanofiber distributions, absence of active substance-polymer agglomeration, and examination of fiber diameters.

**Fig. 12:**
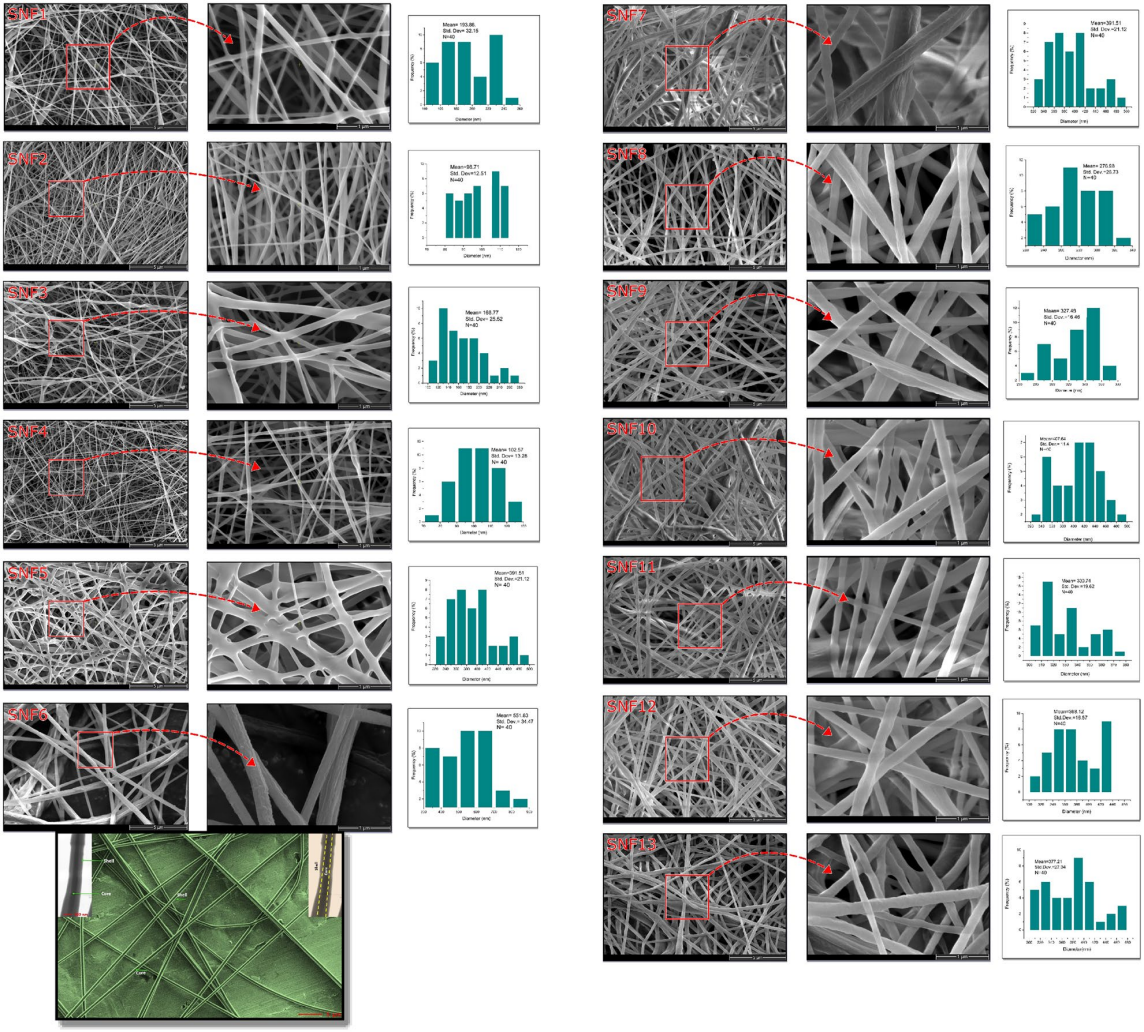
SEM images at 1–5 µm scale of SS/M-SS doped NCM of SNF1- SNF13 with fiber diameter distribution graph; STEM images show the core–shell structure of PCL/PLA/PVA/SS-MSS.

To verify the presence of a core–shell structure, fibers were electrospun directly onto copper grids and then imaged using FE-SEM/STEM (5 µm/500 nm). As shown in Fig. 12, FE-SEM/STEM images clearly reveal the core–shell structure (PCL/PLA shell surrounding a PVA/M-SS core).

SEM images of the coaxially produced fibers with interlocking structures are shown in Fig. 12, within a 1–5 µm range. SNF4 fibers have an average diameter of 70–130 nm. For SS/M-SS doped nanofibers produced using a coaxial nozzle, an increase in fiber diameter was observed, ranging from 300 to 500 nm^21^.

According to the SEM images and their distribution graphs, SNF4 exhibited uniform, fine, and relatively smooth fibers, while SNF5 showed thicker and more irregular fibers with some junctions. Among alkyl substituted amine doped NCMs, SNF6, exhibits the coarsest fibers with the highest diameter and irregular surface features (mean: 551,83 nm). In contrast, SNF9 presents more uniform and thinner fibers. Transitioning from SNF6 to SNF9 indicated a return to finer fiber. As only the aromatic group amine-doped NCMs, SNF10 and SNF13 have relatively close mean diameters, which are 407.64 nm and 368.12 nm, respectively. Finally, the NCMs doped with both aromatic and aliphatic amine groups, SNF11 and SNF12, 330.74 nm, 368.12, respectively, exhibit similar fiber structures and diameter ranges, which are however, SNF12 contains slightly thicker fibers.

#### Cell culture studies of M-SS doped nanofiber composites

Following characterization, the biological activities of the SS/M-SS doped nanofiber mats were investigated. Biocompatibility is crucial for evaluating the suitability of biomaterials for biomedical applications. SS/M-SS showed outstanding cytocompatibility with L929 cells after a 72-h incubation period^99^.

**Fig. 13:**
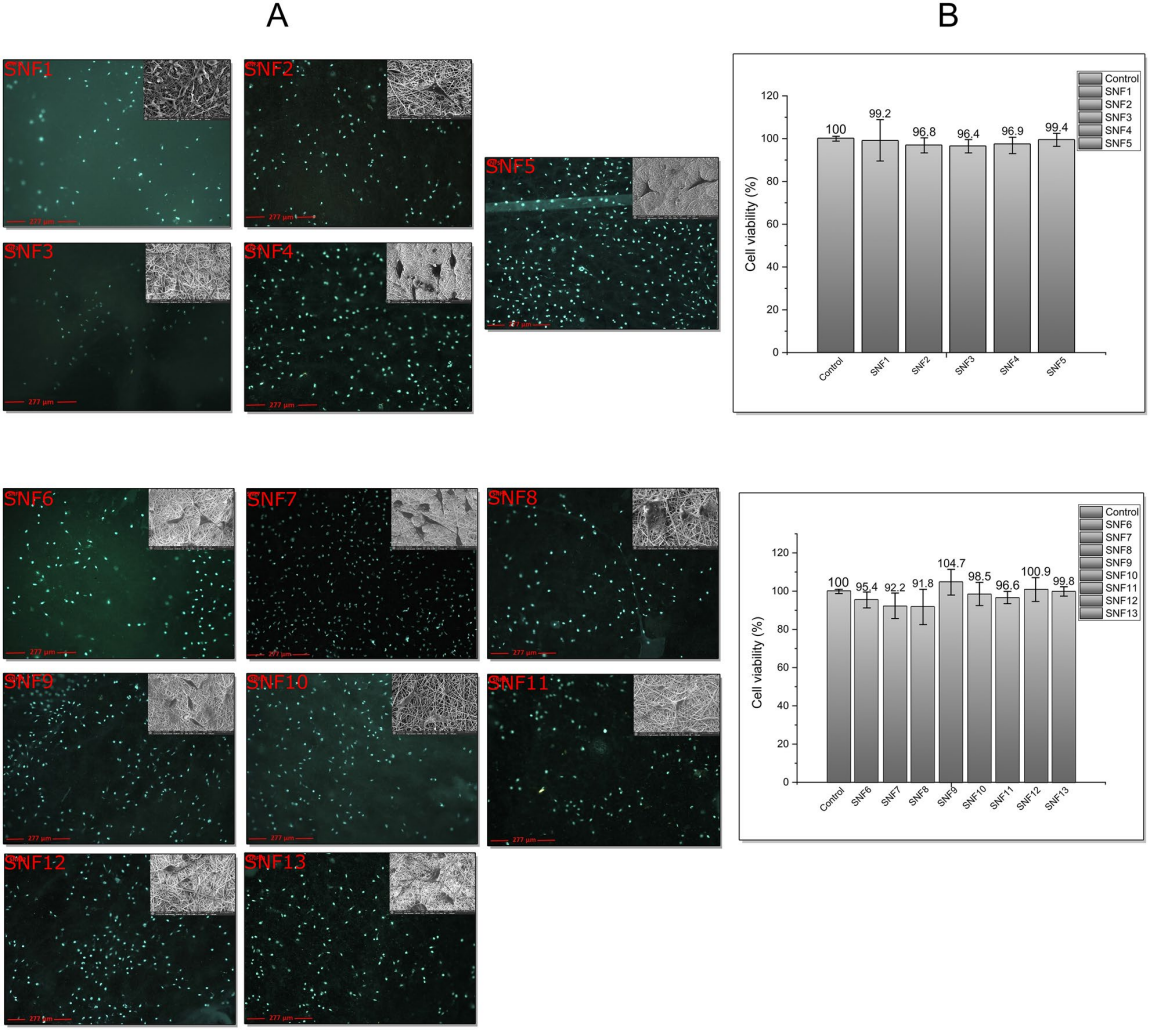
(**A**) Fluorescence microscopy and SEM images of DAPI-labeled L929 mouse fibroblast cells on SNF1-SNF13 nanofiber composite mats. (**B**) Cell viability (%) of L929 cells after 72-h incubation with SNF1-SNF13 NFCs compared to control.

Three distinct concentrations, namely 50%, 70%, and 100% purity, were administered to the L929 cell line, and cell viability was assessed using the MTT assay. The impact of the pure extracts on cell viability is illustrated in Fig. 13.

Figure 13A presents the cell viability results of each polymer forming the matrix (SNF1-SNF3), matrix (SNF4), and SF5/M-SS (SNF6-SNF13) nanofiber composite mats at 72 h. The graph in Fig. 13B shows the cell viability of 100% extracts of the used polymers in pure and composite forms. After a 72-h incubation period, none of the pure extracts reduced the L929 cell line viability below 90% in any of the tested samples. Furthermore, in certain experimental groups, cell viability was observed to exceed 100%.

As indicated in the graph, the SNF5 NCMs exhibited higher cell viability than both the pure polymer forms and the matrix at 72 h. Among the M-SS composites, the highest cell viability was observed in the butylamine-modified SNF9 NCM.

Cytotoxicity (ISO 10993-5 Cell Viability (MTT)) and cell viability tests were conducted^51^. After these tests, SEM imaging of the nanofiber mats was performed, showing cell attachment images at a scale of 20–30 µm. None of the examined mats exhibited cytotoxic effects, and many showed cell viability above 90%. The NCM with butylamine-derived SS (104.7%), SFN9, even exhibited higher cell viability compared to SNF5 NCM (99.4%). The inherent cell adhesiveness of sericins, together with the beneficial properties of M-SS-doped NCMs, demonstrated biocompatibility by supporting cell adhesion and promoting cell growth on the nanofiber mat surface^100^.

In aliphatic groups, viability increased as the carbon chain increased. As the hydrophobicity of the attached functional groups increased, the results of viability tests were also better. Among the alkyl substituent groups, the longest chain butyl amine substituent SS doped NCM, SNF9, showed higher cell viability (104.7%). Both aromatic and aliphatic groups amine doped NCMs gave better results than SNF12 (100.9%) and SNF 11 (96.6%). A similar behavior was observed in only aromatic amine substituent SS containing naphthalene groups. SNF13 (99.8%) gave better results than SNF10 (98.5%). In general, it was observed that cell viability increased when hydrophobicity increased^101^. Only alkyl substituent sericins gave more positive results compared to other groups. The hydrophilic property of SS decreases with the increase in the number of carbons in the alkyl groups. Three main group sericin modifications were made, the most hydrophilic butylamine substituted SS, SNF9, showed the highest value in high cell viability^102^.

## Conclusion

This study focused on modifying sericin through nucleophilic substitution (SN_2_) to replace hydroxyl (–OH) groups with amine derivatives under mild conditions. Para-toluenesulfonyl chloride (p-TsCl), methanesulfonyl chloride (MsCl), and mesitylenesulfonyl chloride were used. Among the sulfonyl chlorides tested MsCl yielded the most effective results due to better reactivity and higher substitution efficiency. FTIR and NMR analyses confirmed the successful conversion of hydroxyl groups into amino groups. Additionally, sulfonyl chlorides reacted with free carboxyl and amino groups, forming amides and sulfonamides, respectively. The amino-modified sericin (M-SS) was electrospun into nanofibers using a coaxial nozzle. While M-SS alone did not form uniform nanofibers, incorporation of polymer additives enabled stable fiber formation, and their compositions were optimized accordingly. All synthesized materials were fully characterized. Biological evaluation, following ISO 10993-5 standards, showed no cytotoxicity. All tested nanofiber mats maintained cell viability above 90%, with some exceeding 100%, indicating enhanced cell proliferation. The highest cell viability was observed in M-SS modified with butylamine. Chemical modification was shown to significantly enhance the biomedical potential of sericin-based materials, particularly for applications in cell culture, drug delivery, and medical devices. Specific modified samples (SNF9, SNF12, and SNF13) demonstrated improved biocompatibility, although further studies are needed to understand the underlying mechanisms. Methylamine-modified sericin displayed the highest tensile strength and crystallization temperature, while the lowest cell adhesion was noted in nanofibers modified with methylamine and ethylamine. All products were produced sustainably and demonstrated strong potential for biological applications due to their biocompatibility and non-toxic nature.

## Supplementary Information

Supplementary Information.

## Data Availability

All data generated or analysed during this study are included in this published article [and its supplementary information files].
