# Supplementary material for: Electrospinning of core shell nanofibers using amine modified sericins
Source: Sci Rep. 2025 Dec 1;15:42836. doi: 10.1038/s41598-025-08984-2 (PMC12669752; doi:10.1038/s41598-025-08984-2)
Supplement: Supplementary file 1 — Supplementary Information. [file 41598_2025_8984_MOESM1_ESM.docx]

**Supplementary Information**

**Modification Of Sericin From Silk Proteins And Production Of Core-Shell Electrospun Nanofibers With PVA/PCL/PVA**

Demet Sezgin Mansuroglu^1,2^, Adem Çınarlı^3*^, Gökhan Çaylı^4^, Demet Gurbuz^3^

^1^Central Laboratory, Kocaeli University, 41001 Kocaeli, Türkiye.

^2^Department of Chemistry and Chemical Processing Technologies, Kocaeli Vocational School, Kocaeli University, 41140 Kocaeli, Türkiye.

^3^Department of Chemistry, Engineering Faculty, Istanbul University-Cerrahpasa, 34320 Avcılar, Istanbul, Türkiye.

^4^Department of Engineering Sciences, Engineering Faculty, Istanbul University- Cerrahpasa, Istanbul, Türkiye.

*email: [adem@iuc.edu.tr](mailto:adem@iuc.edu.tr)

[sezgindemet82@gmail.com](mailto:sezgindemet82@gmail.com), [sezgindemet82@gmail.com](mailto:sezgindemet82@gmail.com), [adem@iuc.edu.tr](mailto:adem@iuc.edu.tr), gokhan.cayli@iuc.edu.tr, demet@iuc.edu.tr

**Table S1.** Amines used in sericin modification and molecular formulas of the products

| Modification  No | Amines added for modification | Amino substutie sericines | Molecular formula of amino substutie sericines | |
| --- | --- | --- | --- | --- |
| 0 | Sericine | _ |  | |
| 1 | Ammonia | Derivative of ammonia-modified sericine (SAMT) |  | |
| 2 | Methylamine | Derivative of sericine modified with methylamine (SMAT) | 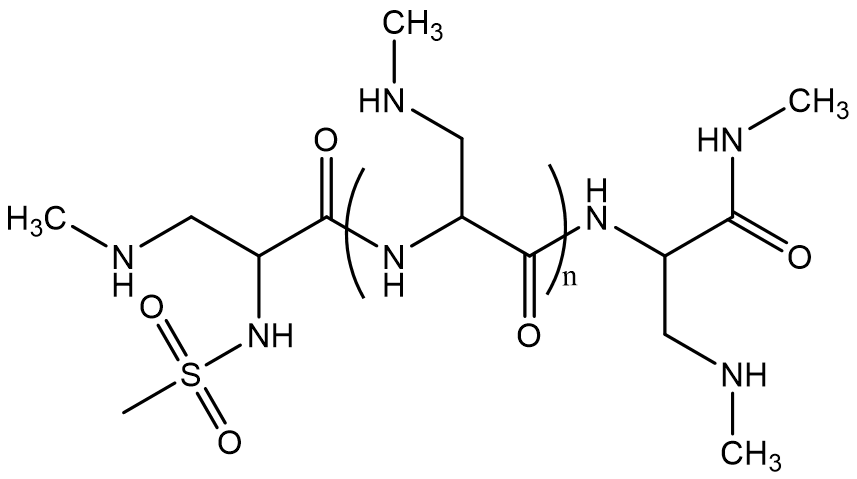 | |
| 3 | Ethylamine | Derivative of sericine modified with ethylamine (SEAT) | 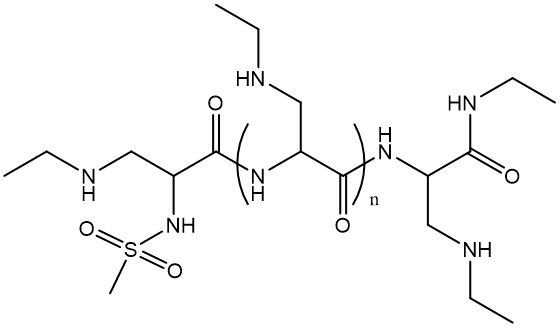 | |
| 4 | Butylamine | Derivative of sericine modified with butylamine (SBUAT) | 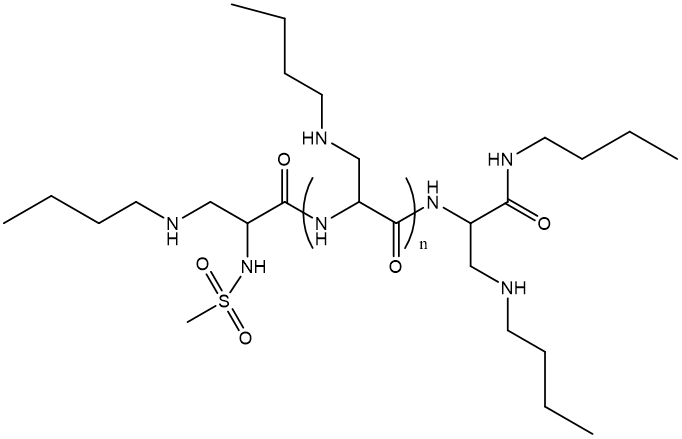 | |
| 5 | Aniline | Derivative of sericine modified with aniline  (SANT) |  | |
| 6 | Benzylamine | Derivative of sericine modified with benzylamine  (SBAT) | |  |
| 7 | Furfurylamine | Derivative of sericine modified with furfurylamine  (SFUAT) | |  |
| 8 | 1- Naphthylamine | Derivative of sericine modified with Naphthylamine (SNAT) | |  |

|  |  |
| --- | --- |
| **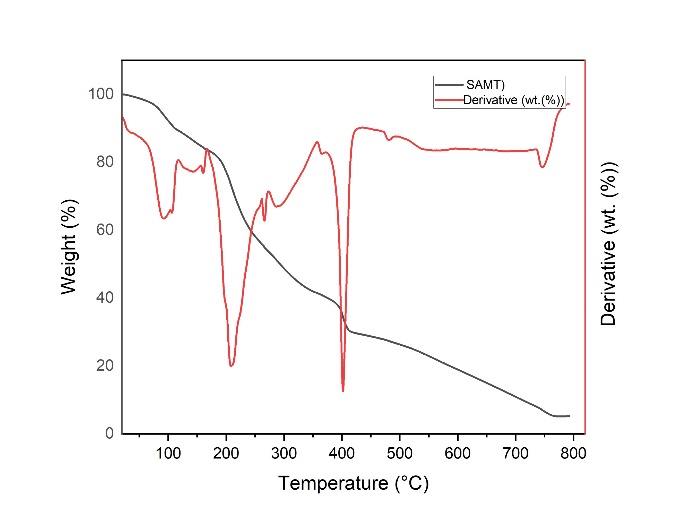** | **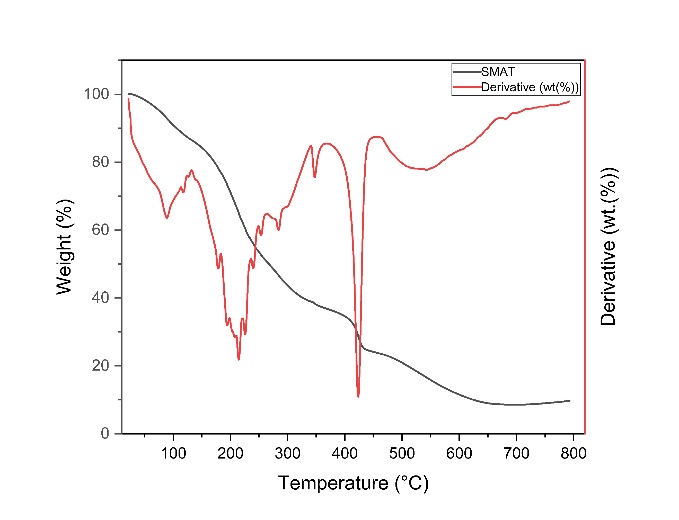** |
| SAMT | SMAT |
| **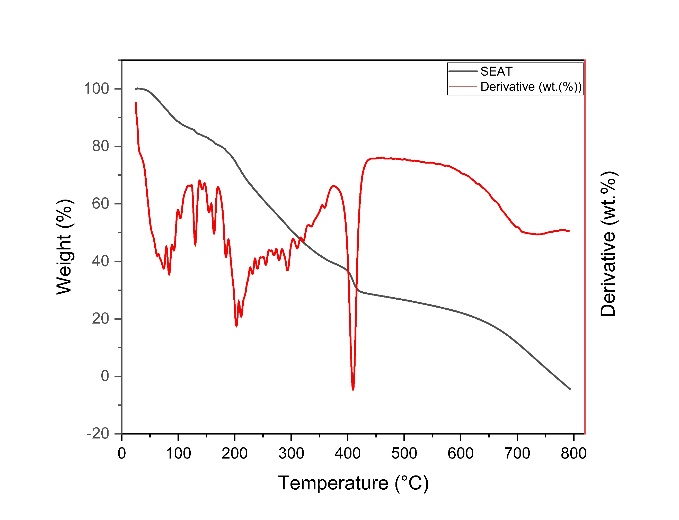** | **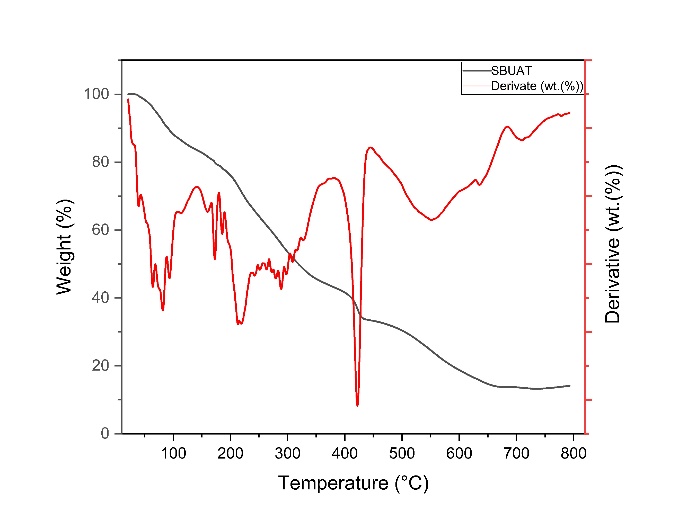** |
| SEAT | SBUAT |
| **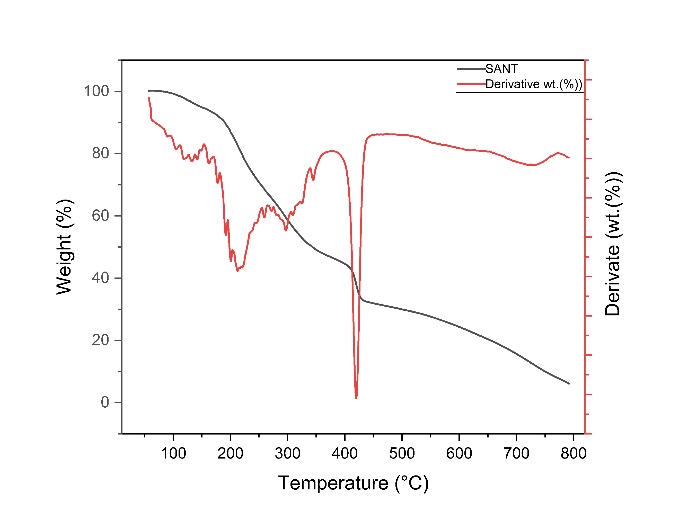** | **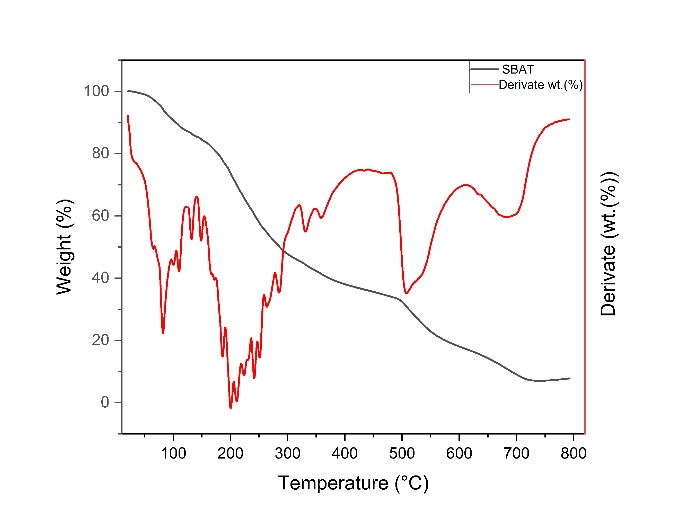** |
| SANT | SBAT |
| **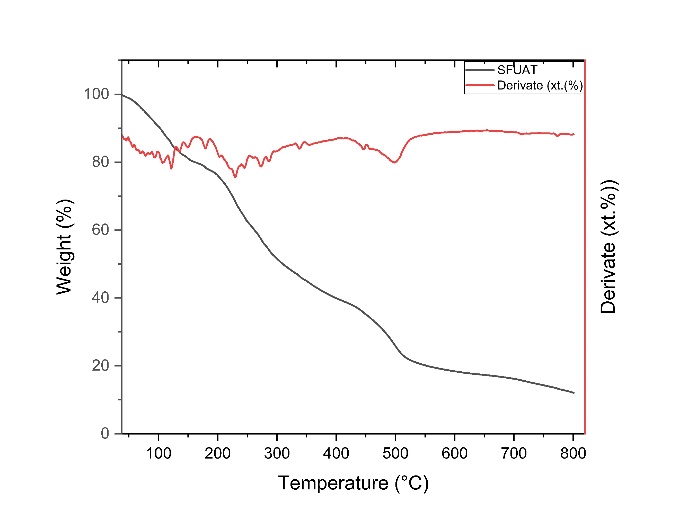** | **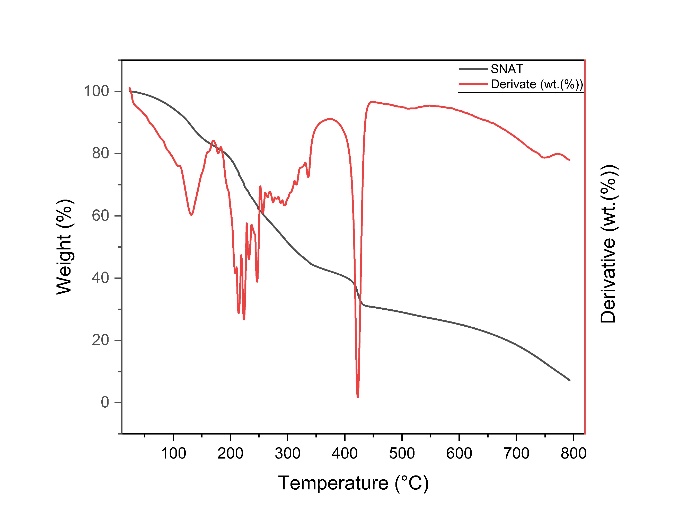** |
| SFUAT | SNAT |

**Fig.S1.** TGA termograms of M-SS

| 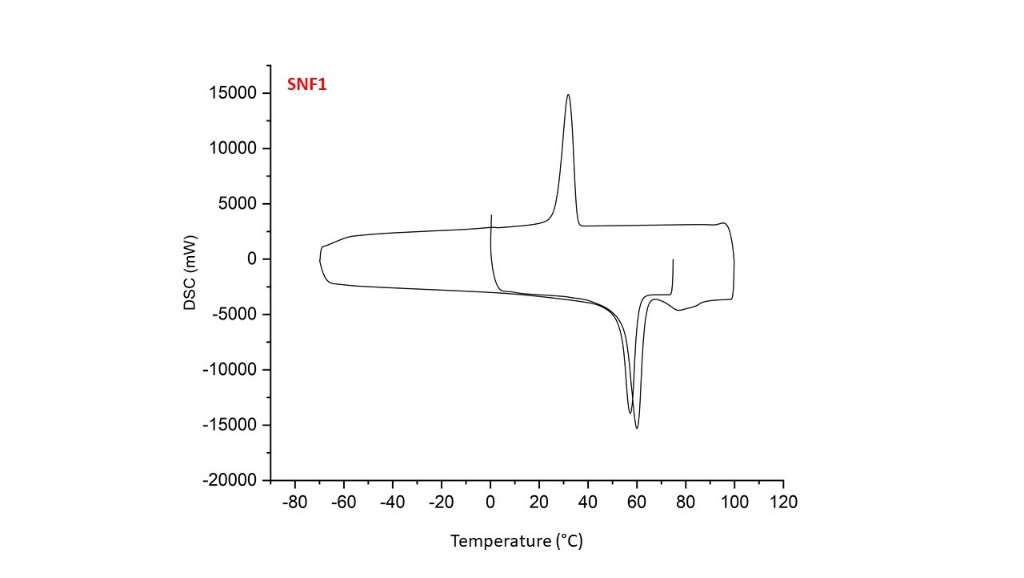 | 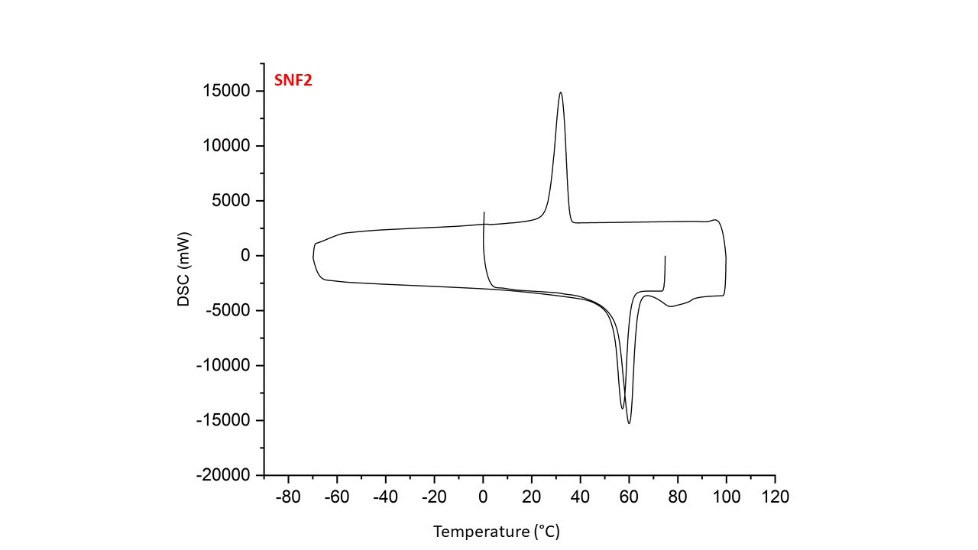 |
| --- | --- |
| 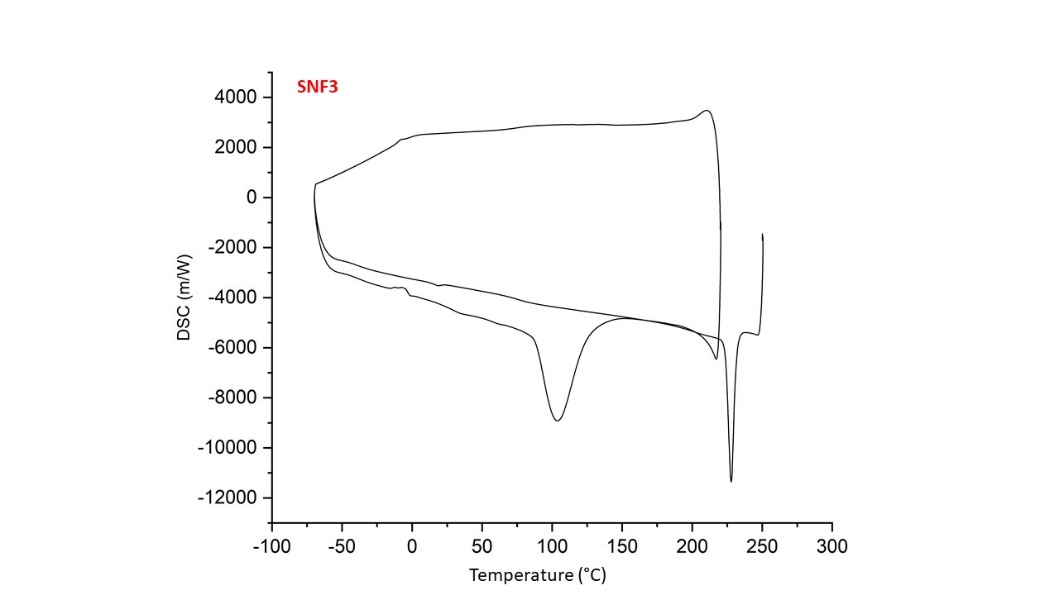 | 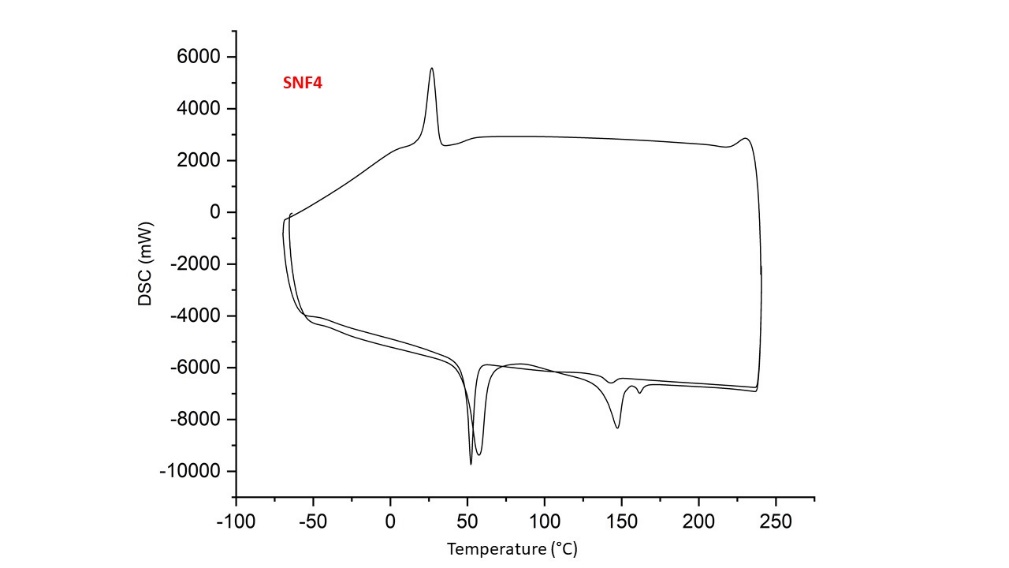 |
| 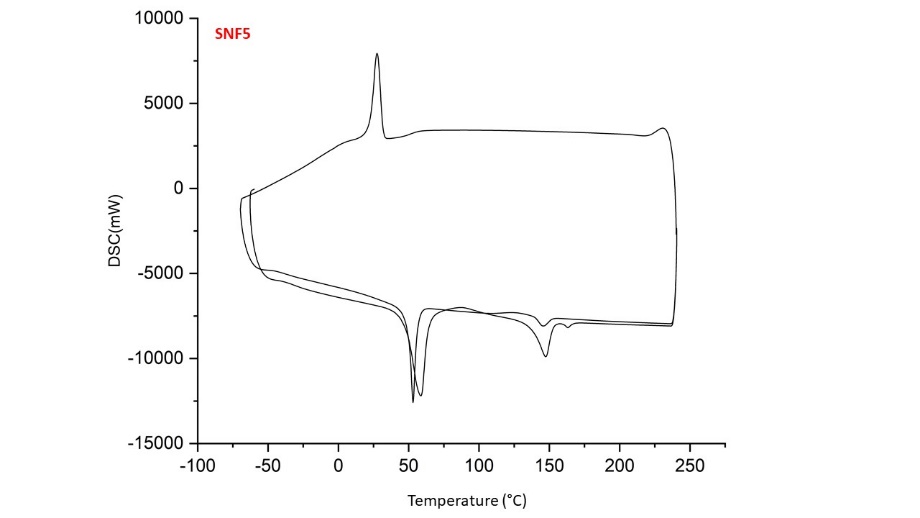 | 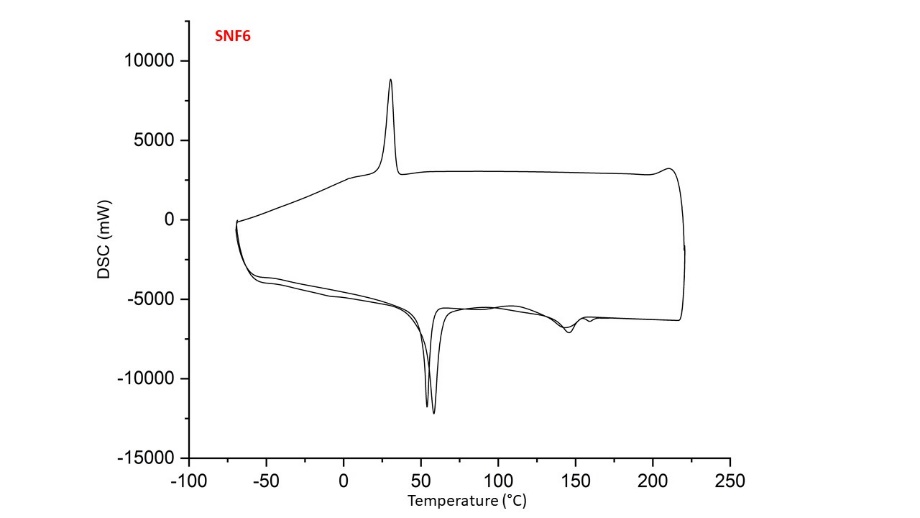 |
| 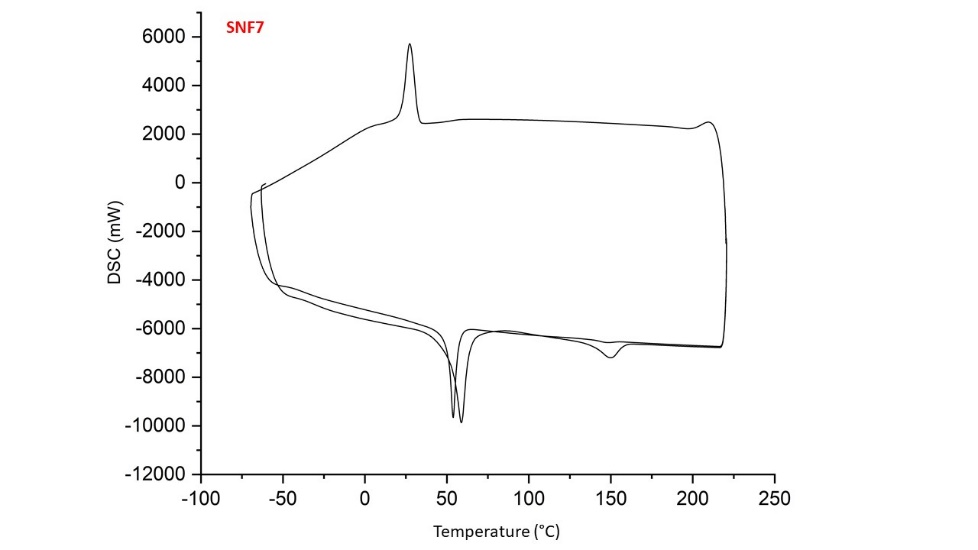 | 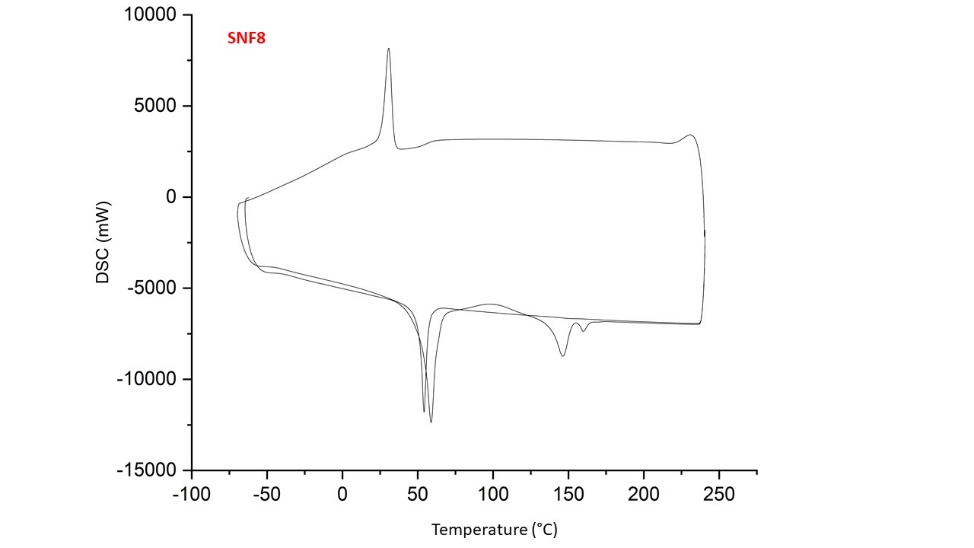 |
| 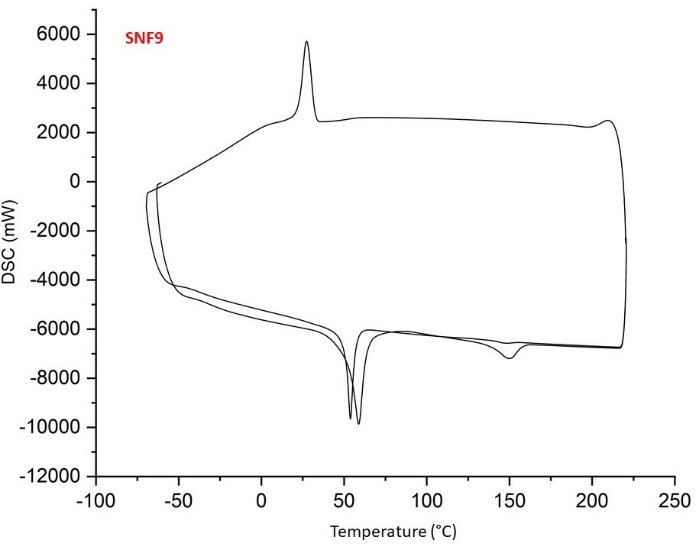 | 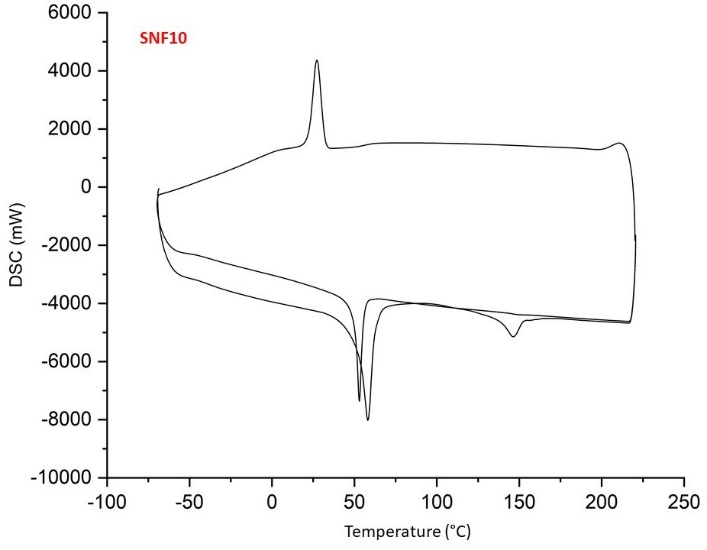 |
| 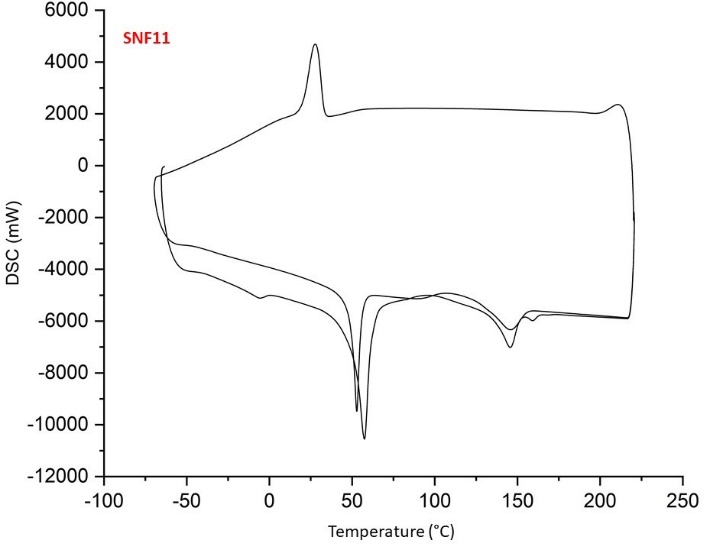 | 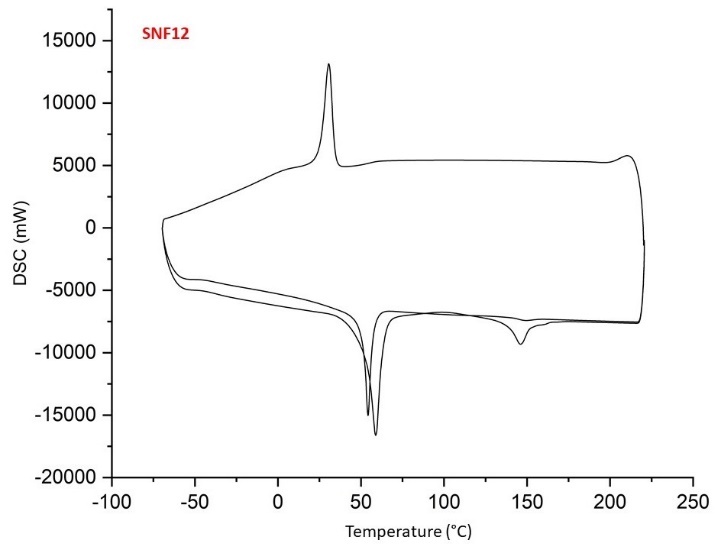 |
| 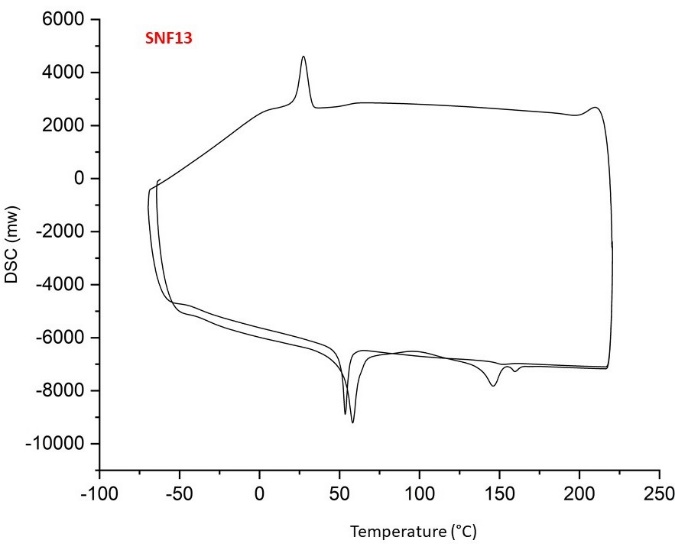 |  |

**Fig.S2.** DSC curves of M-SS Doped nanofiber composites

| 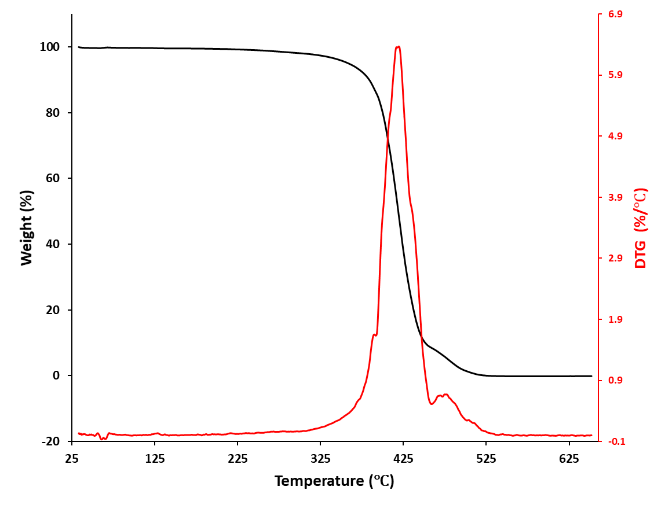 | 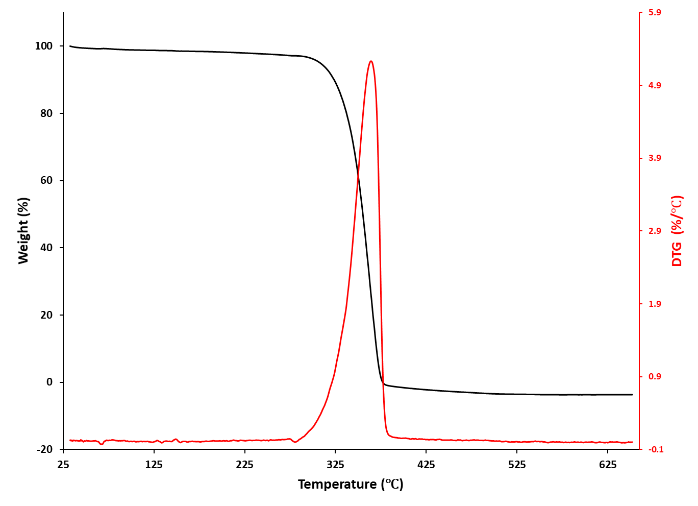 |
| --- | --- |
| SNF1 | SNF2 |
| 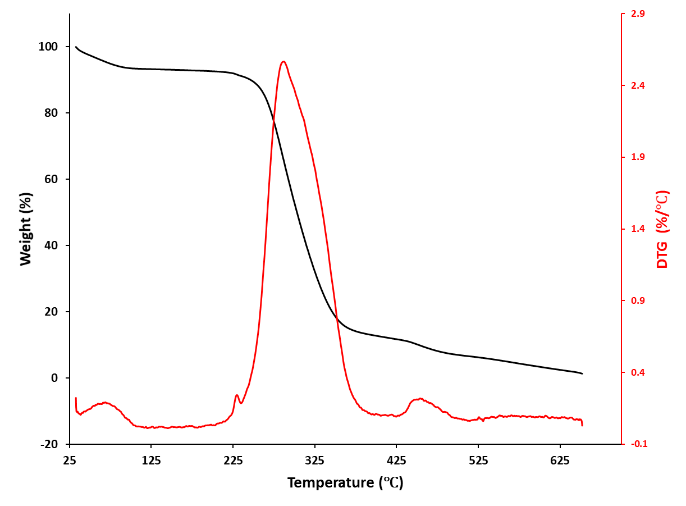 | 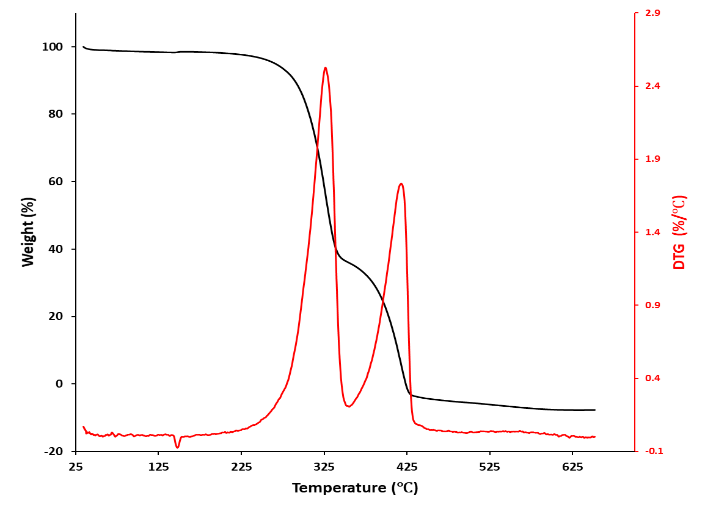 |
| SNF3 | SNF4 |
| 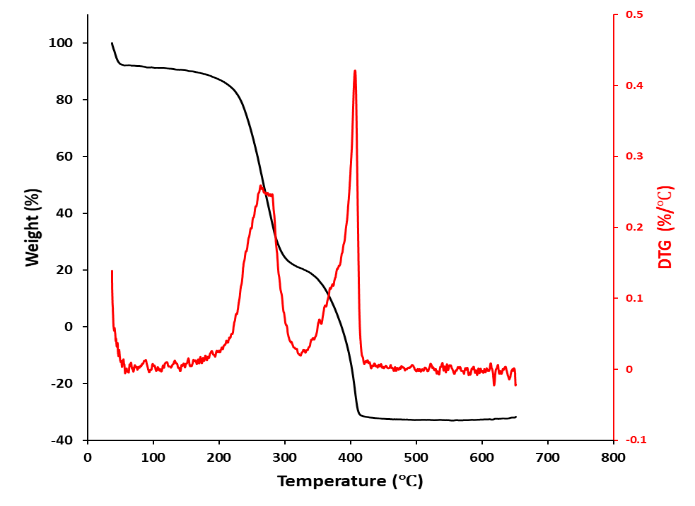 | 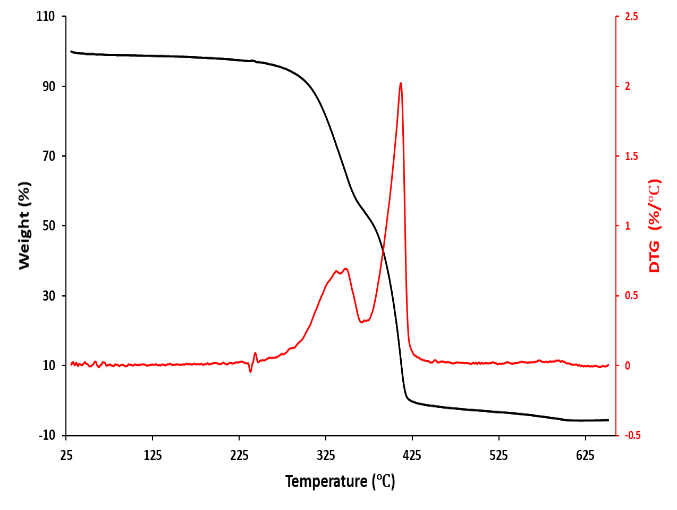 |
| SNF5 | SNF6 |
| 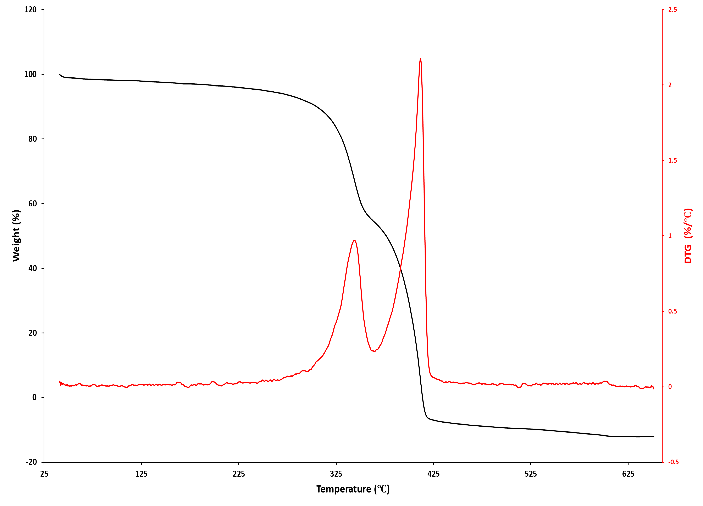 | 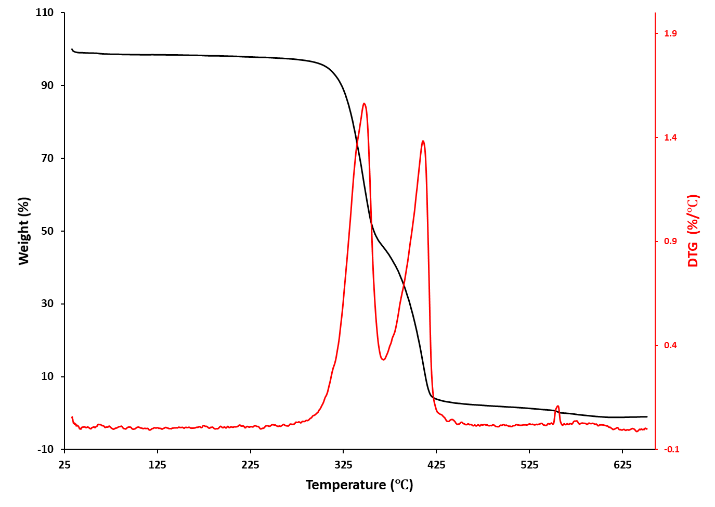 |
| SNF7 | SNF8 |
| 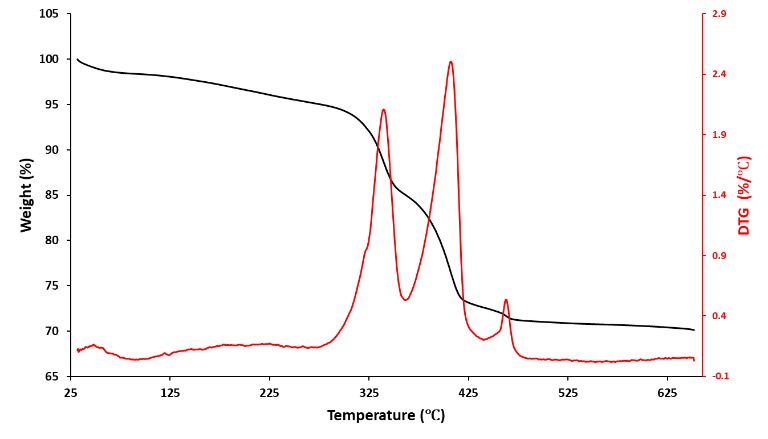 | 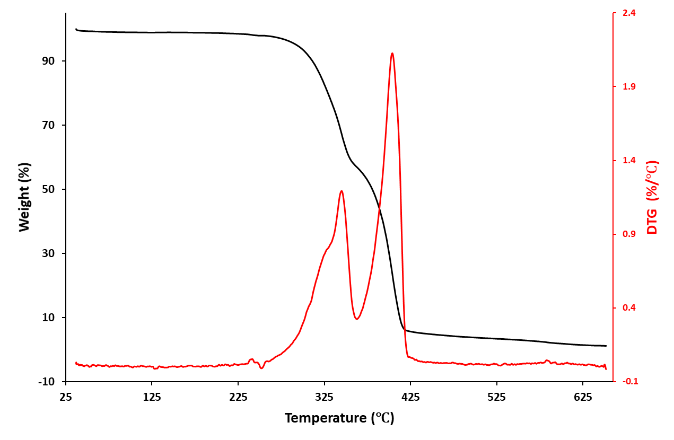 |
| SNF9 | SN10 |
| 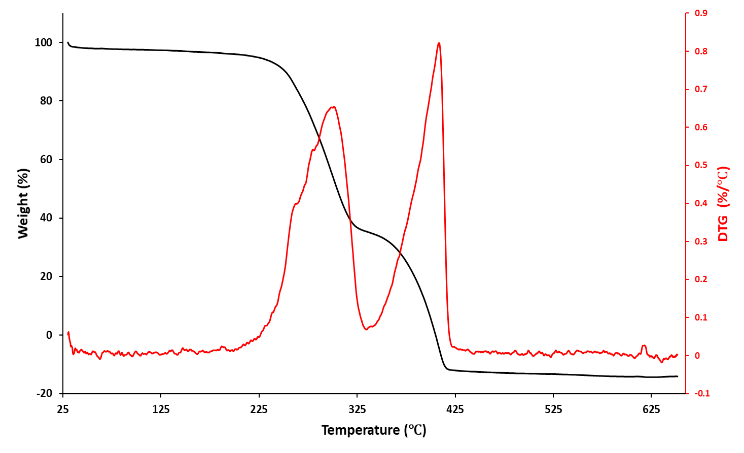 | 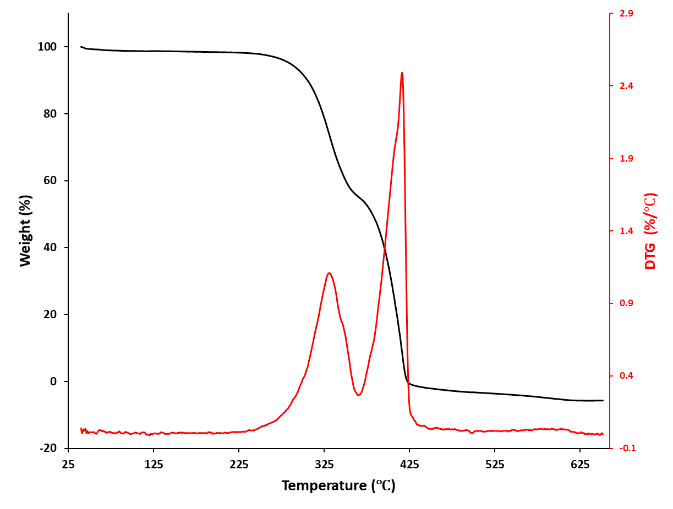 |
| SNF11 | SNF12 |
| 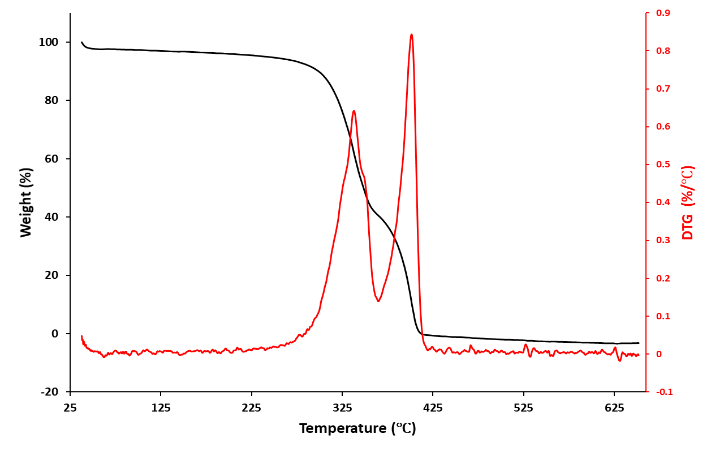 |  |
| SNF13 |  |

**Fig.S3.** TGA termograms of M-SS Doped nanofiber composites


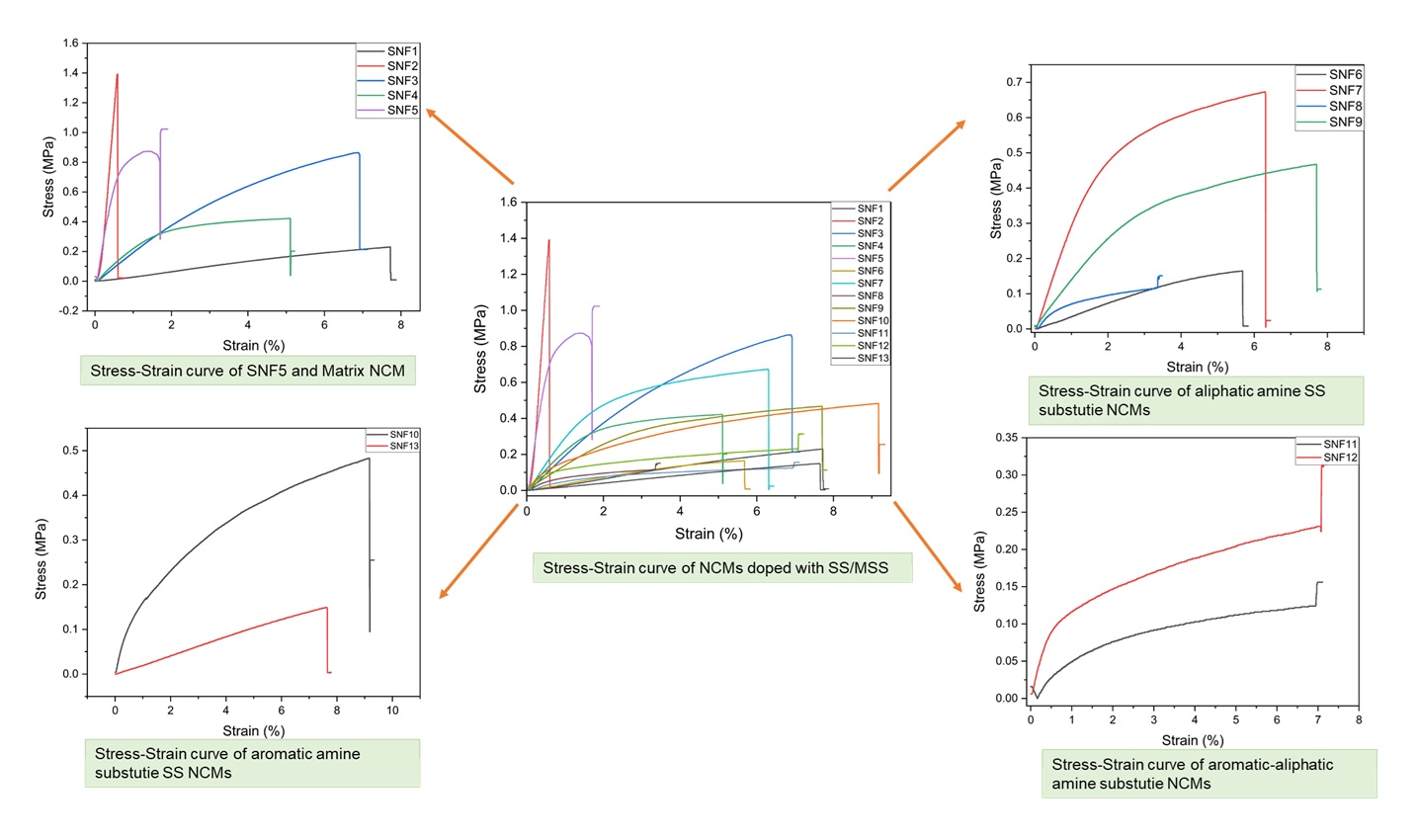


**Fig.S4**  Stress-Strain curve NCMs dopped with SS/M-SS.
